# Prehabilitation of elderly frail or pre-frail patients prior to elective surgery (PRAEP-GO): study protocol for a randomized, controlled, outcome assessor-blinded trial

**DOI:** 10.1186/s13063-022-06401-x

**Published:** 2022-06-06

**Authors:** Stefan J. Schaller, Jörn Kiselev, Verena Loidl, Wilm Quentin, Katrin Schmidt, Rudolf Mörgeli, Tanja Rombey, Reinhard Busse, Ulrich Mansmann, Claudia Spies, on behalf of the PRAEP-GO consortium,, Claudia Spies, Ursula Marschall, Rahel Eckardt-Felmberg, Irmgard Landgraf, Ulrich Schwantes, Reinhard Busse, Ulrich Mansmann, PRAEP-GO investigators, Friedrich Borchers, Rudolf Mörgeli, Eva Schönenberger, Philipp Klassen, Bernadette Kleikamp, Philipp Brandhorst, Anna-Lena H. Spiegel, Bernhard Papenfuß, Jens Dowideit, Caroline Oefele, Volker Gebhardt, Kristina Zappel, Mehmet Gövercin, Thomas König, Claudio Chesi, Anett Reißhauer, Adrian Rosada, Ursula Müller-Werdan, Anja Heymann, Marion Hanke, Jens Leymann, Thomas Saller, Ann-Cathrin Bischof, Angelika Reisner, Wolf Leopold Albrecht, Julia Wojciechowski, Martina Schuldt, Michael Baum, Dijana Brnabic, Björn von Pickardt, Agnieszka Deutschmann, Carsten Scholz, Lars May, Rahel Eckardt-Felmberg, Isabell Wenghöfer, Manfred Blobner, Sima Sattari, Michael Dummert, Carla Nau, Mareike Otto, Ute Voß-Lümers, Danny Lang, Daniel Wiggert

**Affiliations:** 1 Department of Anesthesiology and Operative Intensive Care Medicine (CVK/CCM), Charité – Universitätsmedizin Berlin, corporate member of Freie Universität Berlin and Humboldt-Universität zu Berlin, Chariteplatz 1, 10117 Berlin, Germany; 2 Institute for Medical Information Processing, Biometry, and Epidemiology - IBE, Ludwig-Maximilians-Universität München, Munich, Germany; 3 Department of Health Care Management, Technische Universität Berlin, Berlin, Germany

**Keywords:** Frail elderly, Preoperative exercise, Frailty, Perioperative care, Health services research, Decision-making, Shared, Randomized controlled trial

## Abstract

**Background:**

Frailty is expressed by a reduction in physical capacity, mobility, muscle strength, and endurance. (Pre-)frailty is present in up to 42% of the older surgical population, with an increased risk for peri- and postoperative complications. Consequently, these patients often suffer from a delayed or limited recovery, loss of autonomy and quality of life, and a decrease in functional and cognitive capacities. Since frailty is modifiable, prehabilitation may improve the physiological reserves of patients and reduce the care dependency 12 months after surgery.

**Methods:**

Patients ≥ 70 years old scheduled for elective surgery or intervention will be recruited in this multicenter, randomized controlled study, with a target of 1400 participants with an allocation ratio of 1:1. The intervention consists of (1) a shared decision-making process with the patient, relatives, and an interdisciplinary and interprofessional team and (2) a 3-week multimodal, individualized prehabilitation program including exercise therapy, nutritional intervention, mobility or balance training, and psychosocial interventions and medical assessment. The frequency of the supervised prehabilitation is 5 times/week for 3 weeks. The primary endpoint is defined as the level of care dependency 12 months after surgery or intervention.

**Discussion:**

Prehabilitation has been proven to be effective for different populations, including colorectal, transplant, and cardiac surgery patients. In contrast, evidence for prehabilitation in older, frail patients has not been clearly established. To the best of our knowledge, this is currently the largest prehabilitation study on older people with frailty undergoing general elective surgery.

**Trial registration:**

ClinicalTrials.gov NCT04418271. Registered on 5 June 2020. Universal Trial Number (UTN): U1111-1253-4820

**Supplementary Information:**

The online version contains supplementary material available at 10.1186/s13063-022-06401-x.

## Administrative information

**Table:** 

Title	Prehabilitation of elderly frail or pre-frail patients prior to elective surgery (PRÄP-GO): study protocol for a randomized, controlled, outcome assessor-blinded trial
Trial registration	ClinicalTrials.gov (NCT NCT04418271) Universal Trial Number (UTN): U1111-1253-4820
Protocol version	V1.1 from 13th of June 2020
Funding	Grant 01NVF18024 of the “Innovationsausschuss” of the German Federal Joint Committee (G-BA)
Author details	Stefan J Schaller^1^, Jörn Kiselev^1^, Verena Loidl^2^, Wilm Quentin^3^, Katrin Schmidt^1^, Rudolf Mörgeli^1^, Tanja Rombey^3^, Reinhard Busse^3^, Ulrich Mansmann^2^, Claudia Spies^1^ on behalf of the PRÄP-GO consortium and PRÄP-GO investigators Affiliations: 1 Charité – Universitätsmedizin Berlin, corporate member of Freie Universität Berlin, Humboldt-Universität zu Berlin, and Berlin Institute of Health, Department of Anesthesiology and Operative Intensive Care Medicine (CVK/CCM), Berlin, Germany 2 Institute for Medical Information Processing, Biometry, and Epidemiology - IBE, Ludwig-Maximilians-Universität München, Munich, Germany3 Department of Health Care Management, Technische Universität Berlin, Berlin, Germany
Trial sponsor	Innovationsausschuss beim Gemeinsamen Bundesausschuss Gutenbergstraße 13, 10587 Berlin Postfach 12 06 06, 10596 Berlin E-mail: info@if.g-ba.de The German Federal Joint Committee is a legal entity under public law. Authorized representative: Prof. Josef Hecken Competent supervisory authority: German Federal Ministry of Health
Role of sponsor	G-BA is a public sponsor in Germany. The G-BA had no role in the study design; collection, management, analysis, interpretation, or reporting of the data; report preparation; or publication decisions.

## Introduction

Due to the increasing life expectancy of the population and significant medical progress, more and more complex surgical interventions are performed in older patients. Even though severe postoperative complications, such as mortality, have been reduced in the last decades [1], older patients are at risk of losing autonomy and to develop medium and long-term disabilities in the cognitive and functional domains [2–4]. Additionally, older patients have higher rates of hospital readmissions associated with a delay in functional recovery [5]. The risk of postoperative complications is not only determined by pre-existing conditions, such as diabetes mellitus or cardiovascular disease [6–8] but also by the presence of a frailty syndrome [9–12].

Frailty is associated with restrictions in functional reserves, including mobility, muscle strength, and vital capacity. It is an independent risk factor for the development of postoperative complications, such as postoperative delirium [13], overall mortality, and long-term care [14], as well as long-term cognitive disorders [15]. The prevalence of frailty is estimated to be between 4.0 and 27.3% within the population ≥ 65 years of age [16]. However, the prevalence is much higher in the perioperative setting, with up to 42%, depending on the surgical discipline and screening tool used [17].

Prehabilitation is a term used to describe preventive programs targeting specific health-related issues, and frail patients may benefit from such an approach aiming to reduce the impact of individual frailty syndrome components. Prehabilitation interventions are safe to perform [18, 19] and have been demonstrated to accelerate the recovery of functional skills [20, 21]; reduce rates of postoperative complications, such as delirium and cognitive disorders [22–25]; and shorten hospital stays [26]. However, most of these findings are restricted to specific patient groups or types of surgery, while evidence in a general surgical population is lacking [27].

This investigation aims to evaluate the clinical and health economic effectiveness of prehabilitation in elderly frail or pre-frail patients prior to elective surgery in a multicenter, randomized controlled, outcome assessor-blinded trial, comparing the effectiveness of a prehabilitation program with the standard of care. Patients will be accompanied for a year after surgery, so as to determine the effects of the prehabilitation intervention on long-term care dependency, as well as other factors relating to clinical outcome and economic impact.

## Methods

### Study design

This study is an assessor-blinded, two-arm parallel-group, randomized, controlled, multicenter superiority trial (RCT) in frail or pre-frail patients undergoing elective surgery in Germany with an allocation ratio of 1:1 per hospital and a follow-up period of 12 months. Currently, 19 study centers participate in the recruitment of participants. Updates will be posted on ClinicalTrials.gov and on the project homepage [28, 29].

**Fig. 1:**
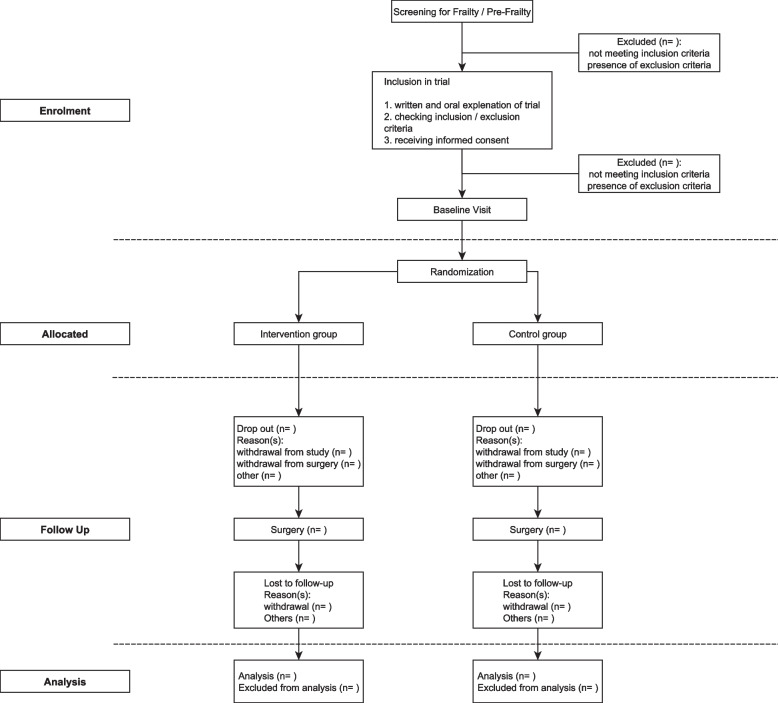
PRAEP-GO flowchart

The intervention group will receive prehabilitation in one of four different settings: (1) inpatient facilities, such as geriatric wards; (2) day clinics; (3) outpatient rehabilitation centers; or (4) home-based via a mobile rehabilitation team. A current list of participating centers and prehabilitation partners, as well as the current version of the study protocol in German and English languages (V1.1; Date: 6/13/2020), can be found online at the project homepage [28]. The flow diagram of the study is presented in Fig. 1, and the SPIRIT Checklist is provided in Additional file 2.

### Objectives and research questions

The objective of this RCT is to evaluate the effectiveness of a 3-week prehabilitation program for patients 70 years of age or older with frailty or pre-frailty undergoing elective surgery.

#### Primary research question

The primary research question is as follows: Can a shared decision-making conference and a 3-week prehabilitation program improve “care dependency” 1 year after surgery?

#### Secondary research questions

The secondary research questions are as follows:

1. Is the planned intervention (a shared decision-making conference and a 3-week prehabilitation program) cost-effective?
2. What is the effect of the intervention within 12 months after surgery on the following parameters: (a) new diagnosis of neurocognitive disorder, (b) frequency of suspected neurocognitive disorder or dementia, (c) frailty status, (d) polypharmacy, (e) alcohol use, (f) tobacco use, (g) sarcopenia, (h) nutritional status, (i) functional status, (j) depression, (k) anxiety, (l) health-related quality of life, (m) disability, (n) fear of falling, (o) incidence of falls, (p) social situation, (q) pain, (r) loneliness, (s) survival, (t) frequency of healthcare resource utilization in the follow-up period, and (u) frequency and length of hospitalizations in the follow-up period.
3. What is the autonomic preference of patients concerning medical decisions and the extent of involvement of patients, relatives, or healthcare professionals in shared-decision making?
4. What is the health trajectory of patients 12 months after surgery with and without a shared decision-making conference and a 3-week prehabilitation program (diagnoses, medication, healthcare resource utilization)?

Additional research questions are explored in an accompanying research program, ANA-PRAEP-GO (NCT04880824), which also includes translational research questions.

### Eligibility criteria

The inclusion criteria are as follows: (1) age ≥ 70 years, (2) planned elective surgery/interventional procedure, (3) expected duration of anesthesia > 60 min, and (4) pre-frail or frail based on Fried’s frailty phenotype, which evaluates muscle strength, gait speed, subjective fatigue, weight loss, and physical activity. Measurements to identify these parameters include hand grip strength and gait speed over 15 ft, self-reported weight loss of 4.5 kg or more within the last 12 months, and self-reported exhaustion within the last week [30]. Metabolic equivalent of task (MET) is used for identifying low levels of physical activity, whereas a MET of < 3 is used as a cutoff (a MET of 3 is defined as moderate activity according to a guideline on activity recommendations in adults [31]). Patients are defined as pre-frail with at least one item indicated as positive and as frail with three or more positive items.

Due to legal reasons relating to the project funding, participation was initially limited to patients of BARMER public health insurance provider. Permission for the inclusion of all patients covered by the German statutory (public) health insurance was obtained in December 2020, and since then, patients from all statutory health insurance companies in Germany have been recruited.

The exclusion criteria are as follows: (1) severe cardiac condition (i.e., New York Heart Association (NYHA) grade IV), (2) severe pulmonary condition (Global Initiative for Chronic Obstructive Lung Disease (GOLD) grade IV), (3) intracranial procedures, (4) palliative patients, (5) language barrier, (6) participation in another interventional trial not approved by the steering committee or another interventional rehabilitation trial, and (7) lack of consent to participate in the study.

### Recruitment and screening

Patients will be recruited during a preoperative visit to the surgical or anesthesia department in each participating study center. All eligible patients that are either frail or pre-frail based on the frailty screening will be invited to participate in the project by a study team physician. Information on goals and duration of the study, the role of each participant, randomization, and any pre-identified risks will be explained in written and oral forms to each potential participant. Written consent to participate is mandatory before any further study-related measures are conducted. Original patient information and consent forms are available in Additional file 3. Enrolled participants are then offered the opportunity to participate in complementary studies, designed to investigate specific research topics in the study population not covered by the grant of this study, including biological samples. Participation in the complementary studies is not required, and all further topics are covered by additional study protocols, are registered as separate research projects in ClinicalTrials.gov, and are subject to additional ethical approval.

### Interventions

The control group will receive no intervention aside from the usual care provided as part of the surgery process and subsequent rehabilitation. The “usual care” comparator appears to be the appropriate option for a pragmatic effectiveness analysis.

The intervention group will receive (1) a shared decision-making (SDM) conference and (2) a 3-week individualized prehabilitation program prior to the elective surgery. Study participation will have no influence on intra- and postoperative processes, such as the scope rehabilitation programs.

#### Shared decision-making (SDM) conference

SDM implies the involvement of the patient in clinical decision-making, based on ethical considerations of informed decisions. Including the patient in the decision-making allows for patient-centered care, i.e., decisions that best suit the individual preferences and needs of the patient rather than the preferences of the healthcare professional [32]. Barriers to participation in the SDM process were identified among older adults, such as physical and mental limitations due to chronic conditions, but also difficulties in providing understandable and clear information to patients regarding their disease and therapy [33, 34].

Traditionally, SDM is organized between a physician and the patient (sometimes with relatives or proxies), while newer approaches involve interdisciplinary or interprofessional teams [35]. PRAEP-GO has adopted both interdisciplinary and interprofessional teams and is based on the three-talk model [36, 37], which consists of three phases (choice talk, option talk, and decision talk). In this study, these three phases will be conducted in different settings. The first phase (choice talk) will take place after the baseline visit and subsequent randomization. The assessor will notify and prepare the patient regarding the next phases, will identify his or her willingness to participate in the decision-making process, and will discuss any needs and priorities that would influence the prehabilitation process. Patients can choose to participate together with a family member or any other person of trust. In the case of a limited (or no) willingness to participate in phase 2 of the SDM process, the assessor will adopt a proxy role for the patient. The patient and assessor will discuss how the assessor can, in his or her role as a proxy, comply with the patient’s needs and priorities. Phase 2 of the SDM process (option talk) is a multidisciplinary and multiprofessional case conference. Mandatory participants of this conference will be physicians from the fields of anesthesiology, geriatrics, and the respective field of the planned surgery or intervention, and either a therapist (physiotherapist or occupational therapist) or a nurse. Additional participation of a general practitioner is encouraged. Participation at the conference is possible through personal attendance or telemedical options, such as video or telephone conferences. During the conference, individual and patient-centered goals of the prehabilitation will be discussed, along with the optimal setting for the prehabilitation. These goals are categorized into strength-, endurance-, mobility-, activities of daily living (ADL)-, nutritional-related, and other interventions. Other goals can include psychosocial and neurocognitive interventions, speech therapy, reduction of polypharmacy, and others, based on the identified needs and goals of the patient. The purposes of this phase are to provide realistic treatment options, with advantages and disadvantages, and to understand the priorities, expectations, and opinions of the patient and healthcare professionals involved. The third and final phase (decision talk) takes place at the end of the SDM conference. The objective of this phase is to define patient-centered goals for the prehabilitation period and establish a comprehensive prehabilitation plan, including a decision on the prehabilitation setting (in-house, day clinic, ambulatory, home-based). For the prehabilitation, one primary goal and two secondary goals are defined; an additional category of “other goals” is included to cover prehabilitation goals that do not fit in one of the former categories (see Table 1). If the patient decided not to participate in the conference, the proposal of the conference will be discussed afterwards.

**Table 1:** Number of interventional sessions per week based on goals. The session duration is 30 min

Goals	Supervised		Unsupervised	
	Week 1	Week 2	Week 3	Weeks 1–3
**Primary**	5	5	5	2
**Secondary 1**	3	2	2	2
**Secondary 2**	1	1	1	1
**Others**	1	2	2	1
**Total**	10	10	10	6

#### Prehabilitation

After completing the SDM process, participants in the intervention group will receive a 3-week prehabilitation. All interventional sessions within the prehabilitation program will be performed by multiprofessional teams with special training in the defined prehabilitation program. During these 3 weeks of personalized prehabilitation, 30 sessions of multimodal therapy intervention of 30 min each will be conducted in the setting selected in the SDM process. As part of the standardization of the intervention, the number of therapy sessions per week is defined based on the goals (primary, secondary, others) for each patient. There will be 10 supervised sessions per week that are performed twice daily on 5 days per week, and the patient is encouraged to do six unsupervised sessions per week, resulting in a total of 45–48 exercise sessions over the course of the prehabilitation period (see Table 1).

Exercises are planned based on the defined goals and the recommendations on physical activity for older adults by the American College of Sports Medicine and the American Heart Association [38–40]: aerobic exercises should be performed on at least three non-consecutive days a week for a minimum of 20 min. Strengthening exercises are to be performed on at least two non-consecutive occasions per week with a minimum of 8 to 10 repetitions, so that each participant should be able to perform 10 to 15 repetitions on each exercise. All exercises are performed with a perceived subjective effort of at least 5 to 6 on a scale between 0 and 10 (0 defined as sitting, 10 as “all-out effort”) [40]. Exercises for increasing mobility and balance performance will be performed based on the Otago Exercise Program (OEP), which has consistently been shown to reduce overall mortality and falls in older individuals [41]. In this trial, the supervising physiotherapist or occupational therapist will teach and supervise the exercise components of the OEP as part of the interventional sessions, and additional unsupervised exercise sessions will be prescribed by the same therapist based on the progress of each participant over the course of the prehabilitation program.

**Fig. 2:**
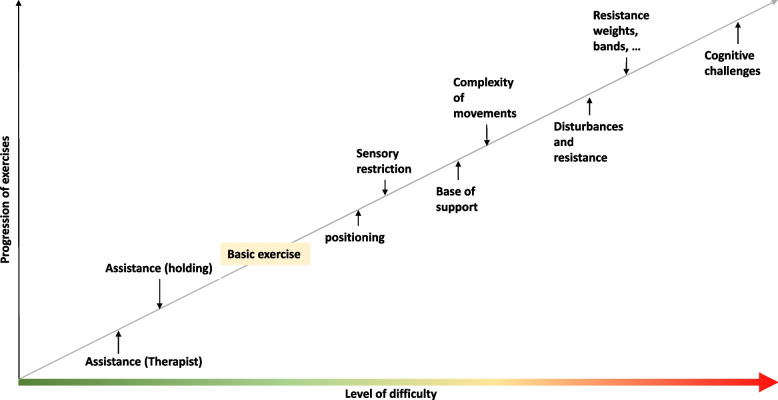
Exercise progression in prehabilitation (adapted from [42])

Using a progression chart, the supervising physical therapist will adapt the performed exercises to comply with the exercise program recommendations over all sessions. The progression chart consists of an adapted version of the published program from Gschwind and Pfenninger [42], who developed an exercise regimen for fall prevention in Switzerland. The underlying principle of this program is to define a basic exercise without any additional input, which can be either assisted or exacerbated to decrease or increase the level of difficulty, as illustrated in Fig. 2. Assistance can be provided either by stabilizing the participant through holding at bars, a chair, or a wall or through assistance by a therapist. Exacerbation can be achieved by changes in body position, limiting sensory input (e.g., closed eyes), combined and complex or asymmetrical movements, disturbances of the movement patterns, and, at the last stage, resistance training with weights, rubber bands, expanders, or other exercise equipment. Combining exercises with cognitive challenges will be used in mobility and balance training and in ADL training.

### Adherence

Adherence will be assessed by reviewing the documentation of the prehabilitation. Additionally, adherence to unsupervised exercises by the participants will be evaluated via a diary, which will be kept by all participants in the intervention group.

### Outcome measures

The primary outcome is the level of care dependency 12 months after surgery. Care dependency is measured using the German structured assessment “Neues Begutachtungsinstrument” (NBA) [43]. The NBA is a standardized tool used by the medical service of the statutory health insurance companies in Germany to assess the need for professional help in the care of older people or people with disabilities, as well as the amount and type of care assistance required. NBA results are a prerequisite for obtaining financial assistance from the insurance for professional care assistance. The NBA consists of six domains: (1) mobility, (2) cognitive and communication ability, (3) behavior and psychological problems, (4) self-dependence, (5) disease-specific demands and burdens, and (6) daily routine scheduling and social contacts. Based on the NBA, six grades of care dependency are defined from zero (no dependency) to five (highest level of dependency) [44].

The following secondary outcome measures are defined:

1. Perioperative complications.
2. Postoperative complications during the hospital stay, such as an unplanned admission to an intensive care unit (ICU), length of stay (LOS) at the ICU, presence of delirium, mobilization speed, and hospital LOS.
3. Exercise adherence and composition during prehabilitation. For this, the responsible therapists will document all exercise and therapy sessions. Patients will fill out a training diary to document all performed unsupervised sessions.
4. Functional ability, measured by the 2-min step test (2-MST) [45], peak expiratory flow (PEF) [46], handgrip strength [47], gait speed over 15 ft [48], stair-climbing speed [49], and Timed Up & Go Test (TUG) [50].
5. Level of mobility and occurrence of falls within 12 months after surgery. Fall incidence will be monitored over the course of all visits. The Life-Space Assessment will be used [51] to evaluate everyday mobility.
6. Health status 12 months after surgery.
7. Psychosocial development of participants, as measured by the PHQ-8 [52], GAD-7 [53], and iADL Questionnaire by Lawton and Brody [54].
8. Healthcare resource utilization within 12 months after surgery. During follow-up, all direct medical and non-medical healthcare-related resource utilization will be monitored using a validated questionnaire for health-related resource use by elderly patients (Fragebogen zur Inanspruchnahme medizinischer und nicht-medizinischer Versorgungsleistungen im Alter (FIMA)) [55, 56].
9. Cost-effectiveness of the prehabilitation program based on cost-utility and cost-effectiveness analyses.

### Study procedures

#### Assessments and study visits

Over the course of the study, participants will be contacted during 17 visits (V2–V18). A short description of each visit is provided in Table 2 and an overview of the assessment categories in Table 3.

**Table 2:** Description of the study visits

Visit	Day	Description
Phase I: Screening and inclusion in the study (intervention and control groups)		
V0	Day 1	Frailty-screening
V1	Day 1/2	Inclusion into study
V2	Day 1/2	Baseline assessment, followed by randomization
Phase II: Shared decision-making (intervention group only)		
V3	V1/V1 + 1–5 days	SDM conference
Phase III: Prehabilitation (intervention group only)		
V4	22–25 days before surgery	Start prehabilitation
V5	V4 + 21 days	End prehabilitation
Phase IV: Follow-up (intervention and control groups)		
V6	7–14 days post-surgery	Discharge from hospital
V7, V8	1/2 months post-surgery	Telemedical/telephone interview
V9	3 months post-surgery	Follow-up at 3 months (telemedical/telephone interview or home visit)
V10–V17	4–11 post-surgery	Telemedical/telephone interview
V18	12 months post-surgery	Follow-up at 12 months (home visit)

**Table 3:** Trial and assessment schedule

Assessments	V0	V1	V2	V3	V4	V5	V6	V7	V8	V9	V10	V11	V12	V13	V14	V15	V16	V17
Frailty status	X																	
Inclusion/exclusion criteria	X																	
Informed consent	X																	
Sociodemographic data		X																
Weight, BMI		X			X			X										X
Medical data		X			X	X	X	X	X	X	X	X	X	X	X	X	X	X
Care dependency		X				X			X									X
Functional assessments		X		X	X													X
Falls		X		X	X	X	X	X	X	X	X	X	X	X	X	X	X	X
Cognitive function		X																X
Depression		X																X
Psychosocial assessment		X							X									x
Autonomy, SDM process			X															
Adherence to prehabilitation					X													
Medical record data			X	X	X	X	X	X	X	X	X	X	X	X	X	X	X	X

#### Assessments

The various assessments to be applied in the different visits are shown in the SPIRIT figure (Additional file 4) and translated study protocol (Additional file 5).

Sociodemographic data consist of age, sex, height, weight, BMI, and level of education. Medical data includes the main and secondary diagnoses, medication, and laboratory values. Medication will be assessed at baseline and during hospital stay, as well as throughout all follow-up visits. Polypharmacy will be identified based on the number of active pharmaceutical agents. This definition is, according to a recent systematic review, the most common definition for polypharmacy [57]. For identifying relevant co-morbidities and their potential impact on surgical outcomes, the Charlson Comorbidity Index (CCI) will be applied [58]. A short questionnaire will assess visual and hearing impairments, with or without assistive devices.

Care dependency will be assessed with the help of two instruments, the aforementioned NBA [43] and the Barthel Index (BI) [59].

Functional assessments include the 2-MST [45], PEF [46], TUG [50], and stair-climbing speed [49, 60]. Additionally, handgrip strength [47] and gait speed over 15 ft [48] will be measured as part of the frailty assessment.

The incidence of falls will be monitored over the entire study period. At baseline, participants will be asked about the number of falls within the last 12 months. The Activities Balance Confidence Scale in its short version (ABC-6) [61] will be used to measure the balance confidence at visits 1, 4, 8, and 17. At each visit following baseline, participants will be asked about any falls since the last visit.

Cognitive function will be screened with the MiniCog™ [62], while additional cognitive tests consist of the Montreal Cognitive Assessment (MOCA) [63] and the Trail Making Test (TMT) parts A and B [64].

Symptoms of depression will be identified with the Patient Health Questionnaire Depression Scale (PHQ-8) [52]. As depression in older people is often linked to anxiety and loneliness, the Generalized Anxiety Disorder Scale-7 (GAD-7) [53] and the UCLA 3-item Loneliness Scale [65] will also be employed.

Psychosocial assessments include the instrumental activities of daily living (IADL) questionnaire by Lawton and Brody [54], the WHO Disability Assessment Schedule 2.0 (WHODAS 2.0) [66], and questionnaires on the social situation of the participant [67] and the EQ-5D-5L for assessing the quality of life (QoL) [68].

The Autonomy Preference Index [69] will be applied before the SDM process begins (intervention group only). After completion of the SDM process, a structured questionnaire [70] will assess the satisfaction of all SDM participants with the course of the process.

Adherence to the prehabilitation program will be monitored through documentation of each session. This documentation includes the goal for each session (e.g., strength, endurance, balance/mobility), intensity and repetitions (strength), or duration (endurance). The Borg Scale will be used to monitor subjective exhaustion during exercises [71]. For unsupervised sessions, a patient diary will be filled out by the patient using the same information. The supervising therapists will ensure the diary is being kept during the supervised prehabilitation sessions.

Peri- and postoperative data will be collected from the hospital electronic database or routine clinical documentation, including duration of surgery and anesthesia, anesthesia method used, peri- and postoperative complications, postoperative therapy, ICU and hospital length of stay, rehabilitation duration, and setting, as well as post-rehabilitation therapies. Additionally, resource utilization, such as contact with physicians and other health professionals, as well as planned and unplanned hospital stays, will be documented using the FIMA Questionnaire [55]. Lastly, emergency room visits (via documentation of participating hospitals, insurance company data, or discharge letters provided by the participants) and mortality will be documented.

### Randomization

After completion of the baseline visit, all participants will be randomized according to a pre-defined protocol. For this purpose, the project statistician will implement an independent, web-based procedure for random group allocation using a randomization tool provided by the Research Electronic Data Capture Software (REDCap) [72]. After randomization by designated study personnel, the patient, the study center, and the trial management team will be informed about the respective allocation.

### Blinding

Due to the nature of the intervention, all participants and staff members in the prehabilitation centers cannot be blinded. Therefore, blinding to group allocation will be limited to outcome assessors. The project coordinator, data manager, and the intervention coordinator will have access to group assignment but will not be involved in assessing participants in the follow-up phase (baseline visit assessments will be conducted prior to randomization).

### Data collection and follow-up

#### Documentation

With the exception of V4 and V5, data from all visits will be recorded directly into the REDCap system. Access to the database is controlled by a password and is granted through the management committee.

The data structure of the database has been designed to ensure a comprehensive and consistent data entry, with controls for range and adequacy, and automated score calculation to minimize errors. Whenever possible, pre-selected options were provided to replace free data texts.

Data quality will be ensured by (a) regular validation of the data by the study team, including checks for comprehensibility, and (b) by re-education visits for all recruitment and prehabilitation centers, if necessary.

Personal information of participants will only be collected after a signed consent form is available. Personal data will include the name, residency, and contact data of all participants. Additional information will include contact data of relatives or significant others, primary care physicians, and care institutions. Due to data safety reasons, this sensitive data will be recorded in a separate REDCap database with a three-factor authentication procedure.

All therapy sessions within the prehabilitation program can be documented directly in REDCap, with a paper-based backup option. Documentation for each session includes goals, performed exercises, duration of the session, intensity, number of sets, and equipment used (e.g., weights). The prescription of unsupervised exercises will be documented additionally. The therapist responsible for each session will provide the documentation.

After completing the study, all gathered data will be stored within the secured data storage system of the leading study center (Charité – Universitätsmedizin Berlin).

#### Patient diary

Each patient will be asked to keep a prehabilitation diary for the documentation of the unsupervised sessions. The diary includes the same items as the form used by the therapists. Completion of the diary will be controlled during the supervised sessions by the therapist working with the patient.

#### Follow-up visits

All participants will be contacted for follow-ups monthly for 12 months after surgery (see Table 2). Telephone interviews (visits 7, 8, and 10 to 17) will cover the incidence of falls within the previous month, the current medication, current pain, and the use of medical, nursing, or therapeutic services. In addition to those items, visits 9, 12, and 15 will assess the quality of life (EQ-5D-5L), disability (WHODAS 2.0), and questions regarding the use of healthcare resources (FIMA).

Three months after surgery, the telephone interview (V9, see Table 2) may be extended to a home or study center visit. In this case, the same assessment as in the final follow-up (V18) will be performed, including all functional performance tests. If the visit cannot be arranged, a shortened test battery will assess all information that can be obtained by telephone.

Visit 18 takes place as a home or study center visit for all included study participants. During this visit, a final assessment of the nursing care dependency level is carried out by means of the NBA, as well as all other assessments initially recorded in visits 0 and 2, so as to identify different trajectories within the two study groups.

Retention of the enrolled participants will be promoted by the monthly telephone interviews, which will serve not only to gather data, but also to allow study personnel to “stay in touch” with the participants.

### Sample size and power considerations

Based on the current literature [73], Table 4 shows the assumed changes in the level of care dependency in the intervention and control groups.

**Table 4:** Quantification of the intervention effect according to Müller-Mai et al

Change in care level	Intervention group	Control group
Improvement of ≥ 1 level	5%	2.5%
No change	47.5%	42.5%
Reduction of 1 level	45%	50%
Reduction of ≥ 2 levels	2.5%	5%
Sum	100%	100%

Formally, the probability of changes in the level of care is an ordered categorical outcome, so the sample size was calculated according to Kolassa [74], using the nQueryAdvisor V7 (MTT2-tmpB3E4 modules; Statsols, Cork, Ireland). Based on this calculation, a sample size of 470 patients in each group will have 80% power to detect the expected quantified effects shown in Table 4, using a Wilcoxon-Mann-Whitney rank sum test with a 0.05 two-sided level. Considering a 2.5% rate of incorrect treatment allocation and 30% loss to follow-up after 12 months, the targeted sample size of the trial is *n* = 1378.

The initial recruitment plan wanted to achieve 1378 participants within a 12-month period. Due to the COVID-19 crisis, the recruitment phase was extended by additional 12 months to a total of 24 months.

### Discontinuing or modifying the allocated intervention (prehabilitation)

All deviations from the intervention protocol will be documented. Possible deviations include, but are not limited to, deviations from the duration of the intervention, as well as changes in the defined goals or setting due to medical or organizational issues, inadequate patient compliance, or drop-out of the study.

### Documentation and reporting of adverse and serious adverse events

Adverse events may occur spontaneously and are expected within our study population, so non-serious adverse events (AEs) (defined as any non-critical and “untoward medical occurrence in a patient or clinical investigation subject administered an investigational intervention”) do not necessarily have a causal relationship to the intervention (adapted from the Note for Guidance on Clinical Safety Data Management: Definitions and Standards for Expedited Reporting (CPMP/ICH/377/95 July 2000)). According to the judgment of the investigator, AEs will be documented as part of the study CRF if an event is considered to be of concern or related to the study or the intervention. Adverse events will be collected from the time of enrollment in the trial until 48 h after the final assessment. Developments will be documented until the event is resolved or explained (max. until day 379 (365 + 14) after elective surgery). The frequency of follow-up is left to the discretion of the investigator.

Serious adverse events (SAEs) are defined as unexpected, fatal, or life-threatening events that are potentially related to the study intervention. An investigator must report the event in the eCRF within 72 h of the first notification. Serious adverse events will be reported up to 48 h after the last study visit, including the following:

- Events occurring during prehabilitation sessions: falls, cardiac arrest, unscheduled hospitalization, unscheduled intervention (e.g., cardiac catheter), or unscheduled surgery
- Events occurring during study visits in the hospital or at home (from the start of the visit up to 48 h after the visit): falls

Insurance is provided to all participants for any adverse event related to study visits or the intervention (including traveling to and from the study/prehabilitation site).

### Statistical analysis plan

Plausibility checks will be carried out prior to any data analyses.

Descriptive analyses of all included patients will be carried out after baseline visits have been completed. Analyses will be carried out for the total sample and stratified by age, gender, group allocation, and frailty status. This is intended to provide a first impression of the data distribution within the strata regarding relevant outcomes. The mean and standard deviation are shown for continuous variables, as well as relative and absolute frequencies for categorical variables.

The primary efficacy endpoint is the change in the level of care dependency—defined as the difference between the care level at baseline and 12 months post-surgery (ΔNBA Score = NBA Score (V18) – NBA Score (V2)).

The prehabilitation effect is defined and tested by a non-parametric approach using the Mann-Whitney-Wilcoxon rank sum test with ties. Moreover, sensitivity analyses will be performed using multivariable logistic regression analysis to adjust for relevant confounders at baseline (e.g., age or gender), as well as to consider longitudinal data and its correlation structure [75].

The secondary will be assessed at different time points (see Table 3, visits 1 to 18) and compared between the intervention and control groups. Moreover, generalized linear mixed-effect regression models will be used, with changes over 1 year as the dependent variable and relevant confounding variables included in the model.

Moreover, sensitivity analyses will be conducted to address the impact of the missingness mechanism, e.g., missing at random or not at random. Complete case analyses will be compared with multiple imputation analyses. Missing data prior to the follow-up measurements might occur (e.g., death, morbidity, loss to follow-up, or withdrawn consent). Death is expected to be a significant source of missing data, and it will be classified as the worst possible endpoint in terms of the level of care dependency.

The principal analyses will be performed according to the intention-to-treat principle and will not be adjusted for screening or baseline covariates or center. The significance level is set to alpha = 5% (two-side). The *p*-value, as a measure of the strength, indicates the association between the dependent and the independent variables. The null hypothesis will be rejected if the *p*-value related to the test statistic for the treatment effect is equal to or smaller than the significance level alpha = 0.05.

The full statistical analysis plan will be finalized and published on the project website after completion of the follow-up assessments, but prior to the commencement of analyses and closing of the database.

#### Interim analyses

There will be no interim analyses.

#### Health economics evaluation

A protocol detailing the methods of the health economics evaluation will be published separately. In brief, the intervention’s cost-effectiveness will be evaluated from the perspective of the statutory health insurance (payer perspective), as well as its cost utility from a societal perspective. Data on resource utilization will be collected using both a bottom-up approach (e.g., patient questionnaires, therapists’ documentation) and a top-down approach (administrative data). The prehabilitation costs (intervention group only) will be calculated using a micro-costing approach, and the costs of healthcare resource use within 12 months after surgery (both groups) will be determined using standard unit costs for the FIMA Questionnaire [76]. Costs will be expressed in euros. Different measures of effectiveness will be considered, including the quality-adjusted life years (QALYs), based on survival data and the patients’ QoL (EQ-5D-5L) scores, as well as appropriate utility weights. Probabilistic and deterministic sensitivity analyses will be performed to explore any uncertainty in the results. A cost-effectiveness acceptability curve will be drawn to determine if prehabilitation is cost-effective under different willingness to pay assumptions.

### Management committee and data safety monitoring committee

A management committee (MC) and a data safety monitoring committee (DSMC) have been established to ensure transparent and safe study practices.

The MC is responsible for developing the study protocol, training of all assessors and healthcare professionals involved in the intervention, and for the initiation of new study centers. Additionally, the MC will monitor the study procedures in all study centers and will ensure adherence to measurement and intervention protocols, as well as ethical guidelines. Deviations from any protocol will be documented and reported.

An independent data and safety monitoring committee (DSMC), consisting of experts in clinical studies, biostatistics, and perioperative and rehabilitation medicine, has been established to monitor recruitment, protocol adherence, and monitoring of follow-up and safety data. Virtual and face-to-face meetings will be regularly conducted, whereas both committees may independently request video conferences. The results of all meetings will be documented in protocols. The DSMC will assess the trial progress and safety, and accumulated trial data will be reported to the DSMC by the study statistician. As a primary responsibility, the DSMC will consider and assess treatment safety (e.g., serious adverse events (SAEs) or deaths). If necessary, recommendations will be made by the DSMC to the MC regarding stopping or continuing the trial. At their discretion, the DSMC may also formulate recommendations relating to the selection/recruitment of participants, their management, improving adherence to protocol-specified regimens and matter relating to patient retention, and procedures for data management and quality control.

The MC will be responsible for promptly assessing any DSMC correspondence or recommendations, to decide whether to continue or terminate the trial, suspend enrolment, or determine whether amendments to the protocol or changes in study conduct are required.

### Dissemination plans

The study report will be submitted for publication to a peer-reviewed medical journal and will be made available via the department’s website. Furthermore, a study report will be submitted to the Federal Joint Committee (G-BA).

A summary report of the final study results will be disseminated to all project partners and on the project homepage. Authorship in scientific publications will adhere to the recommendations of the International Committee of Medical Journal Editors [77].

## Discussion

PRAEP-GO is the first trial to investigate the effect of a shared decision-making conference combined with a 3-week prehabilitation program in a general surgical, (pre-)frail population. Until now, evidence is only available for a variety of specific surgical procedures [27]. Prehabilitation is, according to Wynter-Blyth and Moorthy, “a strategy to begin the rehabilitation process before surgery […]” [78]. Le Roy et al. emphasized the multimodal nature of prehabilitation and recommended the inclusion of (a) physical exercise training, (b) nutritional care, and (c) psychological support as part of the multimodal intervention during prehabilitation [79]. While these definitions offer a general idea of the concept and goals of prehabilitation, a broader and more specific framework on how to design such a program is mostly lacking. This is in part due to the fact that most prehabilitation trials focus on specific patient groups and/or surgical procedures.

Hughes et al. performed a systematic review and meta-analysis on prehabilitation programs before major abdominal surgery. They included 15 RCTs with 907 participants that reported on a variety of abdominal surgical procedures. In this review, prehabilitation leads to a significant reduction in morbidity, but not in LOS or functional recovery [80]. In contrast, Gillis et al. reported a significant reduction in length of hospital stay in a systematic review on prehabilitation, consisting of a nutritional intervention with and without additional exercises before colorectal resection surgery. Three out of nine RCTs that investigated functional outcomes reported a significant improvement [20]. In 2017, another systematic review on prehabilitation prior to abdominal cancer surgery included nine studies, and a total of 549 participants reported mixed results on functional walking capacity, cardiopulmonary fitness, anxiety, postoperative complications, and health-related quality of life. It is worthy to note that the prehabilitation programs varied considerably, with a program duration ranging between 2 and 8 weeks and a variety of therapeutic concepts that included one to three therapeutic modes (diet counseling, physical exercise, and psycho-social support) [81]. However, the physical exercise intervention consisted of walking and/or endurance exercises in all included studies. In another meta-analysis, nine studies with 435 participants receiving abdominal surgery were analyzed. The authors found a significant reduction in morbidity (OR 0.59 [0.38, 0.91] vs. control; OR 0.35 [0.17, 0.71] vs. usual care) and a significant reduction in postoperative pulmonary complications (OR 0.27 [0.13, 0.57] vs. control), but no significant reduction in LOS [82].

Regarding orthopedic surgical procedures, Gometz et al. performed a systematic review on prehabilitation before spinal surgery, including 5 RCTs with 217 participants. While none of the included RCTs reported any significant differences in pain or disability after surgery, 2 of these studies reported a significant reduction in total costs in the prehabilitation groups [83]. Wallis et al. reported on a systematic review and meta-analysis on preoperative intervention before knee or hip joint replacement surgery that included 23 RCTs with 1467 participants. The results of this review suggested that prehabilitation can significantly reduce preoperative pain and, when combined with educational intervention, is able to improve postoperative activity [84]. In another systematic review on prehabilitation before knee or hip arthroplasty surgery, Vasta et al. included 14 studies with 1175 participants. No robust conclusions could be drawn for hip patients; however, despite inconsistent evidence, most studies on knee patients reported postoperative improvements in pain, range of movement, quality of life, and functional scores [85].

For prehabilitation of older persons with frailty or pre-frailty, Milder et al. included 8 studies, with 2 of them ongoing. The six published studies included 168 participants and were highly heterogeneous, although five of the six could demonstrate positive effects, including improved function, reduced complication rates, and reduced mortality [86]. In another systematic review, Baimas-George et al. included 5 studies with 265 participants on prehabilitation in patients with frailty. Again, the included studies were heterogeneous but could demonstrate positive effects regarding LOS, mortality, and distance during 6-min Walk Test [87].

While several trials examined prehabilitation in specific settings, there is still a lack of robust evidence regarding its (cost-)effectiveness in a general surgical, (pre-)frail population.

A novel approach pursued in our trial is to include SDM into the concept of prehabilitation. As part of a European project aiming to identify factors for a successful implementation of integrated care, a mixed-methods study was conducted on the involvement preferences of geriatric patients [33]. While the results suggest that older people in acute need of care had a significantly lower inclination to be involved in a SDM process, qualitative interviews showed that the lack of a proper informational and educational process was at least in part responsible for their reluctance. The SDM process established in PRAEP-GO is based on specific recommendations for implementation of the process with frail older people [88]. To our knowledge, the inclusion of a SDM process into a concept of prehabilitation has not yet been investigated.

In contrast to most other studies in the field of prehabilitation, recruitment in PRAEP-GO is not limited to a single surgical intervention or indication, and the medical exclusion criteria preclude only urgent and intracranial procedures. The primary and many of our secondary endpoints reflect general factors contributing to health, QoL, and patient autonomy, factors that are critical in any evaluation of patient-centered interventions, regardless of the type of intervention.

This approach will result in a cohort that reflects the diversity of routine clinical practice. Being funded by the innovation fund of the Federal Joint Committee, the highest decision-making body of the joint self-government of physicians, dentists, hospitals, and health insurance funds in Germany, the intervention concept will be evaluated and considered for implementation in the routine care of the statutory health insurance funds in Germany.

### Choice of endpoints

Statutory health insurance in Germany generally covers all costs of medical treatments and nursing care assistance [89]. In the latter case, the need for care assistance must be evaluated with the NBA, by a specialized nurse or medical doctor, before care assistance payments can start [42]. In Germany, healthcare costs are based on the principle of solidarity, i.e., the payments of insured individuals are used to cover the costs of the care of those in need [89]. Therefore, statutory health insurance companies in Germany have a special interest, and even the obligation, to minimize costs by implementing preventive strategies [90]. Therefore, the primary endpoint is of particular interest for the German healthcare system, as the NBA was designed not only to evaluate whether a given person needs nursing care, but also if the costs of this care will be reimbursed by the insurance. The NBA assesses the level of care dependency based on reported difficulties in performing activities of daily living. While a validation study was conducted to evaluate the time and effort required for this care based on the different levels of care [91], there are no validation studies examining reliability or the existence of floor/ceiling effects. Nevertheless, the NBA has been selected as the primary endpoint as it enables an effective analysis from the patients’ perspective, with a clear indication of the impact the prehabilitation program can have from a health economic perspective. To compensate for the existing shortcomings of the NBA, several secondary outcomes will be investigated, including functional, cognitive, and psychosocial parameters, as well as outcomes related to medical care such as peri- and postoperative complications, LOS, and post-discharge utilization of the healthcare system.

### Limitations

While the presented protocol has many strengths, some limitations remain. Three potential sources of bias have been identified in our study, which must be discussed.

First, as in all RCTs on therapeutic interventions, a proper blinding of participants and healthcare professionals is not feasible. The PRAEP-GO protocol is limited to an outcome assessor blinding and a blinded statistical analysis.

Second, therapeutic intervention studies lack standardization of the intervention, at least if one defines “standardization” as a rigorous effort to provide the same treatment to all participants. Since a fully standardized intervention is not practicable in therapeutic trials, it is important to provide sufficient information regarding the treatment framework and how the intervention is customized to the needs of the participants. Unfortunately, many therapeutic studies still lack a proper description of their respective intervention [92–94]. In PRAEP-GO, a proper description of the therapy is provided by (a) reporting in detail how an individualized intervention is derived and (b) ensuring that the selected therapies are appropriate to achieve the patient’s goals, which were defined in a standardized process themselves. Additionally, all exercise regimes were based on evidence-based recommendations, such as the American College of Sports Medicine for exercise and physical activity in older adults [38–40].

The third limitation concerns our study population. From a medical perspective, frail elderly patients remain very heterogeneous as a group, which might influence the effectiveness of the intervention. Conversely, a positive evaluation of the study can be assumed to have a high general validity.

## Trial status

The first patient was randomized on 30 June 2020. The trial was paused from 1 November 2020 to 1 March 2021 due to the COVID-19 pandemic. Recruitment will continue until the end of June 2022.

## Supplementary Information

**Additional file 1.** PRAEP-GO investigators.

**Additional file 2.** SPIRIT Checklist.

**Additional file 3.** Model Consent form and information for participants.

**Additional file 4.** Spirit Figure.

**Additional file 5.** Study protocol.

**Additional file 6.** Grant decision letter.

**Additional file 7.** Ethical vote.

## Data Availability

The Institute for Medical Information Processing, Biometry, and Epidemiology of the Ludwig-Maximilian University Munich will handle the randomization as well as the data analysis. The healthcare economic evaluation will be performed by the Department of Healthcare Management of the Technische Universität Berlin. The study database, monitoring, and safety reporting are operated by the Department of Anesthesiology and Operative Intensive Care of the Charité – Universitätsmedizin Berlin. Regularly, safety reports are generated and distributed to the Data Safety and Monitoring Committee. De-identified datasets can be made available on reasonable scientific request to the management committee after the primary publication. Access might be restricted due to German data protection laws.

## Abbreviations

2-MST: 2-min step test
ABC: Activities Balance Confidence Scale
ADL: Activities of daily living
CCI: Charlson Comorbidity Index
DSMC: Data safety monitoring committee
FIMA: Fragebogen zur Inanspruchnahme Medizinischer und nicht-medizinischer Versorgungsleistungen im Alter [English: Healthcare Resource Utilization by the Elderly]
GAD-7: Generalized Anxiety Disorder Scale
G-BA: “Gemeinsamer Bundesausschuss” [English: Federal Joint Committee]
GOLD: Global Initiative for Chronic Obstructive Lung Disease
iADL: Instrumental activities of daily living
ICU: Intensive care unit
LOS: Length of stay
MC: Management committee
MET: Metabolic equivalent of task
MOCA: Montreal Cognitive Assessment
NBA: “Neues Begutachtungsassessment”
NYHA: New York Heart Association
RCT: Randomized controlled trial
SAE: Serious adverse event
SDM: Shared decision-making
OEP: Otago Exercise Program
OR: Odds ratio
PEF: Peak expiratory flow
PHQ-8: Patient Health Questionnaire Depression Scale
QALY: Quality-adjusted life years
QoL: Quality of life
TUG: Timed Up & Go Test
UCLA: University of California, Los Angeles
WHODAS: WHO Disability Assessment Schedule
