# Correction: Prehabilitation of elderly frail or pre-frail patients prior to elective surgery (PRAEP-GO): study protocol for a randomized, controlled, outcome assessor-blinded trial

**DOI:** 10.1186/s13063-023-07106-5

**Published:** 2023-02-14

**Authors:** Stefan J. Schaller, Jörn Kiselev, Verena Loidl, Wilm Quentin, Katrin Schmidt, Rudolf Mörgeli, Tanja Rombey, Reinhard Busse, Ulrich Mansmann, Claudia Spies, on behalf of the PRAEP-GO consortium, PRAEP-GO investigators

**Affiliations:** 1 Department of Anesthesiology and Operative Intensive Care Medicine (CVK/CCM), Charité – Universitätsmedizin Berlin, corporate member of Freie Universität Berlin and Humboldt-Universität zu Berlin, Chariteplatz 1, 10117 Berlin, Germany; 2 Institute for Medical Information Processing, Biometry, and Epidemiology - IBE, Ludwig-Maximilians-Universität München, Munich, Germany; 3 Department of Health Care Management, Technische Universität Berlin, Berlin, Germany

**Correction: Trials 23, 468 (2022)**

**https://doi.org/10.1186/s13063-022-06401-x**

Following publication of the original article [1], it came to the authors' attention that, as a result of the names of the PRAEP-GO investigators not being detailed in the Acknowledgements declaration (they were only provided in additional file 1), the names of these investigators were not showing in PubMed. The article has subsequently been updated to add the names of the investigators to the Acknowledgements.
