# Supplementary material for: Prehabilitation of elderly frail or pre-frail patients prior to elective surgery (PRAEP-GO): study protocol for a randomized, controlled, outcome assessor-blinded trial
Source: Trials. 2022 Jun 6;23:468. doi: 10.1186/s13063-022-06401-x (PMC9167908; doi:10.1186/s13063-022-06401-x)
Supplement: Supplementary file 1 — Additional file 1. PRAEP-GO investigators. [file 13063_2022_6401_MOESM1_ESM.pdf]

The following investigators are contributing to PRAEP-GO:

Charité - Universitätsmedizin Berlin, Berlin, Clinic for Anesthesiology and Operative Intensive Care Medicine (CVK, CCM): Friedrich Borchers, Rudolf Mörgeli, Eva Schönenberger, Philipp Klassen

Charité - Universitätsmedizin Berlin, Berlin, Clinic for Anesthesiology and Operative Intensive Care Medicine (CBF): Bernadette Kleikamp, Philipp Brandhorst

A-K-tiv Therapiezentrum, Eutin: Anna-Lena H. Spiegel

Ambulantes Rehazentrum Ottobrunn, München: Bernhard Papenfuß

AMEOS Krankenhausgesellschaft Ostholstein mbH, Middelburg: Jens Dowideit

Back to Activity München, Munich: Caroline Oefele

BG Klinikum Unfallkrankenhaus Berlin, Berlin: Volker Gebhardt, Kristina Zappel

Brandenburgklinik Berlin-Brandenburg, Bernau: Mehmet Gövercin

Caritas Klinik Maria Heimsuchung Berlin, Berlin: Thomas König, Claudio Chesi

Charité – Universitätsmedizin Berlin, Charité Physiotherapie- und Präventionszentrum, Berlin: Anett Reißhauer

Charité – Universitätsmedizin Berlin, Klinik für Geriatrie und Altersmedizin: Adrian Rosada, Ursula Müller-Werdan

Evangelisches Geriatriezentrum Berlin, Berlin: Ursula Müller-Werdan

evangelisches Krankenhaus Hubertus Berlin, Berlin: Anja Heymann, Marion Hanke

Klinik für Geriatrie Ratzeburg, Ratzeburg: Jens Leymann

Ludwig-Maximilians-Universität München, Munich: Thomas Saller, Ann-Cathrin Bischof

medico concept GmbH Rosenheim, Rosenheim: Angelika Reisner

Physioline München, Munich: Wolf Leopold Albrecht

Physiotherapie Köpenick, Berlin: Julia Wojciechowski

Reha Tagesklinik Berlin Pankow, Berlin: Martina Schuldt

Rehasport Schwabing & Giesing, München: Michael Baum

REHA-Zentrum CAROSSA, Berlin: Dijana Brnabic

Rehazentrum Teltow, Teltow: Björn von Pickardt

Sana Kliniken Sommerfeld, Kremmen: Agnieszka Deutschmann

Sport- & Gesundheitszentrum Königs Wusterhausen, Brandenburg: Carsten Scholz

Sport-REHA GmbH Berlin, Berlin: Lars May

St. Joseph Krankenhaus Berlin, Berlin: Rahel Eckardt-Felmberg, Isabell Wenghöfer

Technical University of Munich, Munich: Manfred Blobner, Sima Sattari

therapiePUNKT München, München: Michael Dummert

University Medical Center Schleswig-Holstein, Lübeck: Carla Nau, Mareike Otto

Vamed Rehabilitationszentrum Lübeck, Lübeck: Ute Voß-Lümers

Vivental Rehabilitation Berlin, Berlin: Danny Lang

Wiggert Physio, Lübeck: Daniel Wiggert
