# Supplementary material for: Prehabilitation of elderly frail or pre-frail patients prior to elective surgery (PRAEP-GO): study protocol for a randomized, controlled, outcome assessor-blinded trial
Source: Trials. 2022 Jun 6;23:468. doi: 10.1186/s13063-022-06401-x (PMC9167908; doi:10.1186/s13063-022-06401-x)
Supplement: Supplementary file 3 — Additional file 3. Model Consent form and information for participants. [file 13063_2022_6401_MOESM3_ESM.pdf]

Studieninformation für Patienten und gesetzl. Vertreter zur Teilnahme an der Besonderen Versorgung *Prähabilitation von älteren Patienten mit Gebrechlichkeitssyndrom vor elektiven Operationen (PRÄP-GO) nach §630a BGB - Randomisiert kontrollierte Multicenterstudie* V1.0 vom 10.03.2020

Mit dieser Studieninformation wollen wir Sie über die Leistungen im Rahmen des Projektes PRÄP-GO informieren, um die Vorbereitung auf Ihre Operation noch besser für Sie zu machen.

Verbesserte Versorgung durch die Erfassung von Frailty

Die Durchführung einer Operation stellt einen großen Stressfaktor für den Körper dar. Dies gilt besonders, wenn bereits vor der Operation bestimmte Risikofaktoren bestehen. Ein solcher Risikofaktor ist die so genannte Gebrechlichkeit, englisch „frailty", die einen entscheidenden Risikofaktor darstellt, der nach einem geplanten chirurgischen Eingriff mit einem erhöhten Komplikations- und Sterberisiko, verlängertem Krankenhausaufenthalt und längerfristigen Einschränkungen der Mobilität einhergeht.

Im Rahmen der vorbereitenden Untersuchung für Ihre Operation wurde festgestellt, dass Sie entweder an einer milden oder ausgeprägteren Form dieser Gebrechlichkeit leiden. Aus diesem Grund möchten wir Ihnen anbieten, an der oben genannten PRÄP-GO-Studie teilzunehmen.

Der Zweck der Studie besteht in der Erfassung von Gebrechlichkeit, um das präoperative Risiko besser einzuschätzen zu können. Patienten ab 70 Jahren sollen im Rahmen dieses Vorhabens bestmöglich für den bevorstehenden operativen Eingriff vorbereitet werden.

Das Hauptziel von PRÄP-GO ist, dass die Wahrscheinlichkeit des Auftretens einer Verschlechterung des Pflegegrades nach einem Jahr reduziert wird. Der Pflegegrad bemisst sich nach den bestehenden Einschränkungen der Selbstständigkeit bzw. der Fähigkeiten des Patienten. Die Berechnung der Pflegebedürftigkeit erfolgt durch die Anzahl von Punkten, die anhand eines detaillierten Fragenkatalogs ermittelt werden.

Wie Sie an PRÄP-GO teilnehmen können

Wenn Sie der Studienteilnahme zustimmen, werden Sie als Patient in Ihrer behandelnden Klinik/Krankenhaus einer von zwei Studiengruppen zugeteilt: der Interventionsgruppe oder der Kontrollgruppe. Die Gruppenzuteilung erfolgt zufällig (randomisiert) wie beim Werfen einer Münze. Dies ist wichtig, um später möglichst unverfälschte Schlussfolgerungen ziehen zu können; die Studienärzte oder die behandelnden Ärzte haben keinen Einfluss auf die Gruppenzuteilung.

Die Teilnehmer der Kontrollgruppe werden nach dem klinischen Standard weiter behandelt und zusammen mit den Teilnehmern der Interventionsgruppe für 12 Monate postoperativ nachbeobachtet. Für die Teilnehmer der Interventionsgruppe findet die Operation nach der gezielten und individuell abgestimmten interdisziplinären dreiwöchigen „Prähabilitation" statt. Der Begriff Prähabilitation steht – analog zur Rehabilitation – für den Aufbau von Kraft, Beweglichkeit und Ausdauer bereits vor einem Eingriff. Das 3-wöchige Prähabilitationsprogramm der Interventionsgruppe wird in einer Shared Decision-Making (SDM) Konferenz gemeinsam mit dem Patienten (ggf. mit Einverständnis des

Patienten auch ein Angehöriger) Ärzten verschiedener Fachrichtungen (Anästhesie, Chirurgie, Geriatrie) sowie einem Physiotherapeuten, Ergotherapeuten oder einer Pflegefachkraft festgelegt. SDM bedeutet „Partizipative Entscheidungsfindung". Dies ist eine Form der Kommunikation zwischen Arzt und Patient. Der Ansatz sieht vor, Informationen auszutauschen und eine Entscheidung gemeinsam und gleichberechtigt mit Blick auf die nachfolgende Behandlung zu finden – auch im Rahmen von Televisiten mit Videoübertragung.

Die therapeutischen Inhalte der Prähabilitation sind Physiotherapie, ggf. zusätzlich Atemtraining, Ernährungsberatung, Überprüfung der Dauermedikation, Anämie-Behandlung, Logopädie, neurokognitives Training und Ergotherapie. Die Interventionsgruppe erhält nach der Prähabilitation den geplanten operativen Eingriff nach klinischem Standard.

Zusätzlich soll das Projekt PRÄP-GO Ihre Versorgung auch nach der Krankenhausentlassung verbessern. Wesentliche Leistungen von PRÄP-GO sind daher auch die Nachuntersuchungen, die bis 12 Monate nach Ihrer Entlassung aus der Kooperationsklinik meist einmal im Monat telefonisch aber auch im Rahmen zweier Visiten (3 Monate und 12 Monate) in der Klinik oder bei Ihnen zu Hause erfolgen. Ziel dieser Nachuntersuchungen ist das frühzeitige Erkennen von gesundheitlichen Beeinträchtigungen und die Messung unseres Hauptzielparameters, des Pflegegrades.

Vor der Einwilligung zur Teilnahme an der Studie ist insbesondere Folgendes zu bedenken

Im Rahmen der PRÄP-GO-Studie wird durch geschultes Studienpersonal festgestellt, ob bei Ihnen körperliche und/oder psychische/kognitive Beeinträchtigungen vorliegen. Die Erhebung dieser Beeinträchtigungen orientiert sich an der Pflegegraderhebung des Medizinischen Dienst der Krankenkassen (MDK). Wir möchten Sie darauf hinweisen, dass der durch das Studienpersonal erhobene Pflegegrad nicht zu einem rechtlichen Anspruch aus Leistungen der Pflegeversicherung führt und dieser auch von Ihrer bisherigen Einstufung abweichen kann. Das Ergebnis der Erhebung wird von uns weder an Ihre Krankenkasse, an den MDK oder die Pflegekasse übermittelt, noch in Ihrer Krankenakte dokumentiert. Sollten Sie eine Überprüfung Ihres Pflegegrades wünschen, müssen Sie sich selbst an den MDK wenden und von diesem ein entsprechendes Gutachten einfordern. Wir wollen damit die methodische Qualität (externe Validität) unseres Vorhabens sichern und populationsbasierte Forschung fördern, die valide und objektive Ergebnisse liefert. Zudem wollen wir Ihnen ermöglichen, unabhängig und motiviert an unserer Studie teilzunehmen. Auf Anfrage erläutern wir Ihnen gerne die Gesamtergebnisse der Studie, sobald diese vorliegen.

Teilnahme und Widerruf

Sie erklären Ihre Teilnahme durch Ihre Unterschrift auf der Teilnahmeerklärung. Sie können Ihre Teilnahmeerklärung innerhalb von zwei Wochen nach Ihrer Abgabe widerrufen. Der Widerruf muss schriftlich oder zur Niederschrift bei der Charité erklärt werden. Die Angabe von Gründen ist nicht erforderlich. Durch den Widerruf der Teilnahmeerklärung wird Ihre Teilnahme an PRÄP-GO rückwirkend beendet. Leistungen aus PRÄP-GO können Sie dann nicht mehr in Anspruch nehmen.

Nach Ablauf der Widerrufsfrist sind Sie bis zur Durchführung der letzten Nachuntersuchung an Ihre Teilnahme gebunden. Danach endet Ihre Teilnahme automatisch. Während der Bindung können Sie Ihre Teilnahme vorzeitig aus einem wichtigen Grund beenden, z.B. aufgrund einer nachhaltigen Störung des Vertrauensverhältnisses zu Ihrem Arzt. Die Teilnahme kann auch durch die Studienleitung von PRÄP-GO beendet werden, wenn hierfür wichtige Gründe vorliegen, wenn z.B. ein bestehendes Ausschlusskriterium nachträglich festgestellt wird.

Sollten Sie sich gegen eine Teilnahme an PRÄP-GO entscheiden, entsteht Ihnen kein Nachteil in der weiteren Behandlung. Ihnen stehen selbstverständlich die Leistungen über die Regelversorgung zur Verfügung.

Ihre Teilnahme endet in jedem Fall, sobald das Projekt endet. Es entstehen Ihnen oder Ihrer Versicherung **keine** zusätzlichen Kosten. Alle Kosten sind über das Projekt gedeckt.

Ihren Widerruf richten Sie bitte an Ihren behandelnden Arzt:

Bitte geben Sie dabei das Vertragskennzeichen **01NVF18024** sowie den Vertragsnamen „PRÄP-GO“ an.

Versicherungsschutz

Für Schäden beim Transport per Taxi oder öffentlichen Verkehrsmitteln zu den Nachuntersuchungen liegt im Rahmen dieser Studie eine Wege- Unfallversicherung vor.

Die Studienärzte und -mitarbeiter sind durch die Betriebshaftpflichtversicherung der Klinik gegen Haftungsansprüche, welche aus ihrem schuldhaften Verhalten resultieren könnten, versichert.

Studieninformation für Patienten und gesetzl. Vertreter zur <u>Datenverarbeitung</u> im Rahmen der Besonderen Versorgung *Prähabilitation von älteren Patienten mit Gebrechlichkeitssyndrom vor elektiven Operationen (PRÄP-GO)-* Randomisiert kontrollierte Multicenterstudie V1.0 vom 10.03.2020

Das Wichtigste vorab: Der Datenschutz wird von der Charité und unseren Kooperationspartnern sehr gewissenhaft eingehalten.

Bei datenschutzrechtlichen Fragen wenden Sie sich bitte zunächst an Ihren behandelnden Arzt (Name, Telefonnr.): ____

Sollte dieser Ihnen nicht weiterhelfen können, haben Sie jederzeit die Möglichkeit sich telefonisch (030 / 450 63 12 45),

per FAX (030 / 450 755 10 64) oder

E-Mail: projekt-praep-go@charite.de an die PRÄP-GO-Projektleitung an der Charité zu wenden.

Die Verantwortung für die studienbedingte Erhebung personenbezogener Daten liegt bei Univ.-Prof. Dr. med. Claudia Spies, Klinik für Anästhesiologie mit Schwerpunkt operative Intensivmedizin CCM/CVK, Charité – Universitätsmedizin Berlin, Campus-Virchow-Klinikum, Augustenburger Platz 1, 13353 Berlin.

Bei Anliegen zur Datenverarbeitung und zur Einhaltung der datenschutzrechtlichen Anforderungen können Sie sich auch an die Stabsstelle Datenschutz der Charité wenden:

Stabsstelle Datenschutz
Charité – Universitätsmedizin Berlin
Charitéplatz 1
10117 Berlin
Tel. +4930450580016
E-Mail: datenschutz@charite.de

Folgende Daten werden für die Durchführung von PRÄP-GO von dem Kooperationskrankenhaus verarbeitet:

Die Klinik, in der Sie behandelt werden, erhebt klinische Basisdaten und Ihre Behandlungsdaten im Rahmen der Routine-Datenerhebung. Bei den klinischen Basisdaten handelt es sich um Gesundheitsdaten aus dem geriatrischen Assessment (Bewertung), die vor der Operation erhoben werden. Hierzu gehören beispielsweise die Altersgruppe, das Geschlecht, die Körpergröße, das Körpergewicht, sowie Gesundheitsdaten zu Ihrer kognitiven, emotionalen, funktionellen, und medizinischen Leistungsfähigkeit sowie zu Ihrer sozialen Situation. Die erhobenen Daten werden zur Verbesserung der Patientensicherheit und der Versorgungsqualität gemäß PRÄP-GO benötigt und sind elementare Grundlage für die Durchführung einer Entscheidungsfindungs-Konferenz (auch als SDM (Shared Decision Making)-Konferenz bezeichnet), die auch telemedizinisch unterstützt werden kann. Sie bilden außerdem die Grundlage der Festlegung Ihrer individuellen Therapieziele, falls Sie der Interventionsgruppe, die eine Prähabilitation erhält, zugelost werden.

Rechtsgrundlage für die Verarbeitung der klinischen Basisdaten ist Ihre Einwilligung. Die Einwilligung ist freiwillig und jederzeit mit Wirkung für die Zukunft widerrufbar. Die Rechtsgrundlage für die zur Durchführung des Behandlungsvertrags erforderlichen Daten ergibt sich aus dem jeweiligen Landeskrankenhausgesetz. Die Kooperationsklinik bewahrt Ihre Daten, die sich aus der Behandlung in

diesem Krankenhaus ergeben, für einen Zeitraum von 10 Jahren auf, anschließend werden die Daten gelöscht bzw. papierbasierte Daten werden vernichtet.

Folgende Daten werden für die Durchführung von PRÄP-GO von der Charité – Universitätsmedizin Berlin verarbeitet:

Abrechnung

Damit die beteiligten Kooperationspartner eine Vergütung für ihre Leistungen erhalten, müssen sie eine Abrechnung erstellen und Ihre dazu notwendigen Daten, wie etwa Name, Geburtsdatum, Art der Inanspruchnahme, Behandlungstag, dokumentierte Leistungen und Diagnosen, an die Charité übersenden. Bei der Charité werden die Abrechnungsdaten auf Richtigkeit geprüft. Rechtsgrundlage für die Verarbeitung der Abrechnungsdaten ist Art. 6 Abs. 1 lit. a) DS-GVO, Art. 9 Abs. 2 lit. a), DS-GVO.

Daten im Rahmen der Televisite

Die Charité unterstützt die Klinik, in der Sie behandelt werden, bei der Festlegung der Komponenten der Prähabilitation (Mitbehandlung). Hierzu werden die von Ihnen in der Kooperationsklinik erhobenen medizinischen Basisdaten an die Charité übermittelt, dort ausgewertet und für die Teilnehmer der SDM-Konferenz aufbereitet. Bei telemedizinischer Unterstützung wird die Software CGM ELVI verwendet, die auch Ihnen eine telemedizinische Teilnahme ermöglicht. Um Ihre bestmögliche Versorgung zu gewährleisten, werden die Daten, die von Ihnen an der Charité vorliegen, für die Visite de-pseudonymisiert. Diese de-pseudonymisierten Daten werden von der Charité nur zu Mitbehandlungszwecken in der Videokonferenz verwendet.

Das telemedizinische System selbst besteht aus einer zertifizierten Software zur Durchführung von Audio/Video-Konferenzen., welche die strengen Sicherheitskriterien des Bundesmantelvertrages „Vereinbarung über die Anforderungen an die technischen Verfahren zur Videosprechstunde gemäß § 291g Absatz 4 SGB V“ erfüllt. Es werden keine Gesprächsdaten aufgezeichnet oder gespeichert.

Gesundheitsdaten zur Studiendurchführung

Sofern Sie einwilligen, werden die nach der Basisdatenerhebung und in der Videokonferenz erhobenen Daten/Protokolle von der Charité mit den Studiendaten aus der Klinik und Daten aus Ihren Follow-up-Visiten, die bis ein Jahr nach Krankenhausentlassung stattfinden, gespeichert und verarbeitet, um so Rückschlüsse auf die Wirksamkeit der im Rahmen von PRÄP-GO durchgeführten besonderen Versorgung ziehen zu können. Insofern werden Gesundheitsdaten verarbeitet, die Rechtsgrundlage hierfür ist Ihre Einwilligung. Die Einwilligung ist freiwillig und jederzeit mit Wirkung für die Zukunft widerrufbar.

Im Rahmen der studienbedingten Erhebung möchten wir Sie bitten, wenn Sie in einem kurzen geistigen Leistungstest (MiniCog™) Auffälligkeiten zeigen, eine ausgiebigere Untersuchung Ihrer kognitiven Leistungsfähigkeit mithilfe von Übungen auf einem I-Pad zu 3 Zeitpunkten (präoperativ, 3 Monate und 1 Jahr) und weitere Befragungen vorzunehmen. Die kognitive Untersuchung und die Befragungen werden ca. 1,5 h in Anspruch nehmen.

Ihre im Rahmen der kognitiven Leistungstests pseudonymisiert mittels Cantab-Connect-Software ermittelten Studiendaten werden bei Cambridge Cognition, Tunbridge Court Bottisham, Cambridge, CB25 9TU, UK sicher gespeichert. Das Vereinigte Königreich ist ein sogenanntes Drittland, für das noch kein Angemessenheitsbeschluss der Europäischen Kommission über ein der Europäischen Union vergleichbares, angemessenes Datenschutzniveau vorliegt. Es bestehen auch noch keine geeigneten Garantien im Sinne der Datenschutz-Grundverordnung. Gegenwärtig wird die Übermittlung der Daten noch als zulässig eingestuft. Sollte bis zum Dezember 2020 kein Angemessenheitsbeschluss getroffen sein, ist die Rechtsgrundlage für die Übermittlung Ihre Einwilligung. Alle CANTAB Connect Studiendaten werden zwischen den Tablets synchronisiert und durch sichere Cloudserver der Firma Armor (armor.com oder Telefon: 1-844-682-2858) im Auftrag von Cambridge Cognition zentralisiert. Zugriff erhält das Studienteam der Charité zur Extraktion der Daten und Überführung in die Studiendatenbank auf sicheren Charité-Servern. Zusätzlich erhalten Mitarbeiter des Berliner Institut für Gesundheitsforschung (BIH) die anonymisierten Ergebnisse Ihrer kognitiven Testungen sowie

anonymisierte personenbezogene Daten von Ihnen wie klinische Kovariablen (Geschlecht, Bildung, Ausbildungsjahre, höchster Bildungsabschluss, Muttersprache, Einnahme psychotroper Substanzen (regelmäßig/ in den letzten 3 Monaten vor der Testung; welche, Bestehende chronische Erkrankungen, allgemeine Erkrankung; Neuropsychiatrische und/oder neurologische Diagnose (alternativ: „Sind oder waren sie in der Vergangenheit wegen neuropsychiatrischer (z. B. Depression, Angststörung, Schizophrenie,..) oder neurologischer Störungen (z. B. kognitive Beeinträchtigungen, Demenz, Schlaganfall, Parkinson,..) in Behandlung? Welche?“), da diese als Lizenzgeber das Cloud-System mitverwalten. Mithilfe dieser Daten entwickeln das Berliner Institut für Gesundheitsforschung (BIH) und die Clinical Research Unit CCM/CVK/CBF/CBB, Campus Virchow Klinikum, Augustenburger Platz 1, 13353 Berlin und die Klinik für Anästhesiologie mit Schwerpunkt operative Intensivmedizin CCM/CVK eine gemeinsame deutsche Normdatenbank für 14 Testungen von CantabConnect.

Zusätzlich werden weitere Gesundheitsdaten und weitere Daten über den Verlauf nach der Operation (Komplikationen, Verweildauer im Krankenhaus) nach §21 KHEntgG (Gesetz über die Entgelte für voll- und teilstationäre Krankenhausleistungen – Krankenhausentgeltgesetz) sowie Routinedaten aus Ihrer Krankenakte zur Erforschung der Frailty zu Studienzwecken entnommen. Ebenso werden Verlegungsbriefe, Entlassbriefe und bundeseinheitlicher Medikationsplan an die Charité übermittelt. Dafür entbinden Sie den behandelnden Arzt von seiner Schweigepflicht.

Falls wir Sie nicht erreichen, würden wir die Behandlungsdaten bei Ihrem Hausarzt oder ihrem Angehörigen und aus Ihrer Klinik erheben und diese ausschließlich für den Zweck der o.g. Studie verwenden. Autorisierte Studienmitarbeiter würden eine Anfrage an das jeweilige Meldeamt (Melderegisterauskunft) zu Ihrem Verbleib nach §§ 44, 45 Bundesmeldegesetz am Studienende vornehmen, sofern wir Sie nicht erreichen können. Die Daten werden benötigt, um Langzeitschäden und Mortalität im Zusammenhang mit der Erkrankung Gebrechlichkeit zu erfassen. Diese Daten werden ebenfalls in der Datenbank des Studienleiters zur Auswertung im Rahmen der Studienfragestellung zusammengeführt.

Die Charité speichert Ihre für den Studienzweck erhobenen Gesundheitsdaten in einem elektronischen Programm namens REDcap. Der Server mit der Datenbank basierend auf REDCap (Research Electronic Data Capture, ein etabliertes Werkzeug zur Erhebung von Daten im wissenschaftlichen Bereich; https://projectredcap.org) befindet sich im Rechenzentrum der Charité – Universitätsmedizin Berlin.

Rechtsgrundlage für die Datenverarbeitung durch die Charité ist Ihre Einwilligung. Die Einwilligung ist freiwillig und jederzeit mit Wirkung für die Zukunft widerrufbar. Die Charité löscht Ihre Daten, sobald sie diese nicht mehr benötigt. Das ist der Fall, wenn Ihre Daten zum Studienzweck abgeglichen wurden, also spätestens dann, wenn die Behandlung beendet ist und gesetzliche Aufbewahrungsfristen (10 Jahre) abgelaufen sind.

Daten zu Leistungsausgaben

Sofern Sie einwilligen, werden die Sie betreffenden bei Ihrer Krankenkasse gespeicherten Leistungsausgaben des Zeitraumes der Studienteilnahme an die Charité übermittelt. Dies ermöglicht, die klinischen Outcome-Daten der Studienteilnehmer standardisiert zu erheben. Zu den Leistungsdaten gehören die Kostendaten zu ärztlichen und zahnärztlichen Behandlungen, zu Arznei-, Heil- und Hilfsmitteln, zu Krankhausbehandlungen sowie zu Rehabilitationsleistungen, aber auch alle anderen Leistungsausgaben). Insofern werden Gesundheitsdaten verarbeitet, die Rechtsgrundlage hierfür ist Ihre Einwilligung. Die Einwilligung ist freiwillig und jederzeit mit Wirkung für die Zukunft widerrufbar.

Folgende Daten werden für die Durchführung von PRÄP-GO von den teilnehmenden Prähabilitationsanbietern verarbeitet:

Sofern Sie einwilligen, werden die im Rahmen der Prähabilitation erhobenen Gesundheitsdaten, die zum einen Ihre physische und psychische Verfassung, aber auch Angaben zu den durchgeführten Übungen und damit in Zusammenhang stehende Informationen betreffen von dem jeweiligen Prähabilitationsdienstleister erhoben und an die Charité übermittelt. Insofern werden Gesundheitsdaten verarbeitet, die Rechtsgrundlage hierfür ist Ihre Einwilligung. Die Einwilligung ist freiwillig und jederzeit

mit Wirkung für die Zukunft widerrufbar. Die Rechtsgrundlage für die zur Durchführung des Behandlungsvertrags erforderlichen Daten ergibt sich aus der Datenschutz-Grundverordnung und dem Bundesdatenschutzgesetz. Der Prähabilitationsdienstleister bewahrt Ihre Daten, die sich aus der Behandlung ergeben, für einen Zeitraum von 10 Jahren auf, anschließend werden die Daten gelöscht bzw. papierbasierte Daten werden vernichtet.

Folgende Daten werden für die Durchführung von PRÄP-GO von den teilnehmenden Kooperationskliniken und der Charité verarbeitet:

Für die SDM-Konferenz wird mit Ihnen besprochen, ob Sie eine Teilnahme daran wünschen, um zeitgerecht einen Termin zu koordinieren.

Für die Besprechung im Rahmen der SDM-Konferenz werden Basisdaten (u.a. Vitaldaten und Medikation) erhoben und an die Charité übermittelt. Mit Ihrer Einwilligung kann auch Ihr SDM-Protokoll, das die von Ihnen in der Videokonferenz erhobenen Daten enthält, zur Unterstützung der einrichtungs- und sektorübergreifend organisierten Nachbehandlung weitergeführt und genutzt werden. Bei den Daten handelt es sich um Gesundheitsdaten. Rechtsgrundlage für die Datenverarbeitung ist Ihre Einwilligung. Die Einwilligung ist freiwillig und Sie können diese jederzeit mit Wirkung für die Zukunft widerrufen. Ihre behandelnde Klinik löschen Ihre Daten, sobald sie diese nicht mehr benötigen. Dies ist der Fall, wenn Ihre Daten zur Durchführung des Behandlungsvertrags nicht mehr erforderlich sind und gesetzlich vorgeschriebene Aufbewahrungsfristen (bis zu 30 Jahren) abgelaufen sind.

Rechtsgrundlage für die Datenverarbeitung durch die Kooperationspartner ist Ihre Einwilligung. Die Einwilligung ist freiwillig und jederzeit mit Wirkung für die Zukunft widerrufbar. Die Charité und die Kooperationspartner sind bei der Datenverarbeitung zur Einhaltung sämtlicher Datenschutz-bestimmungen verpflichtet. Dies gilt auch nach Beendigung Ihrer Behandlung. Die im Rahmen dieser Studie aufgezeichneten Daten und Analyseergebnisse werden ohne Ihren Namen ermittelt, wissenschaftlich ausgewertet und den gesetzlichen Bestimmungen entsprechend mindestens 10 Jahre archiviert.

Datenverarbeitung durch weitere Dritte

Der externe Evaluationspartner Univ. Prof. Dr. Ulrich Mansmann, Direktor des IBE, Ludwig Maximilians-Universität München verarbeitet Ihre Daten für den Zweck der Evaluierung der besonderen Versorgungsform in pseudonymisierter Form. PD Dr. Alexander Geissler, Dipl.-Ing. TU Berlin Fakultät Wirtschaft & Management, ITM, Management im Gesundheitswesen, verarbeiten Ihre Daten zur ökonomischen Bewertung der besonderen Versorgung in pseudonymisierter Form. Rechtsgrundlage für die Datenverarbeitung ist § 25 Abs. 3 LKGBln. Die Daten werden gelöscht, sobald sie zur Erreichung des Forschungszwecks nicht mehr erforderlich sind, sofern keine gesetzlichen Aufbewahrungspflichten entgegenstehen.

Widerruf der Einwilligung in die Datenverarbeitung

Die Datenverarbeitungen, für die keine gesetzliche Erlaubnis vorliegt, sind nur zulässig, soweit Sie in die jeweilige Datenverarbeitung eingewilligt haben. Wenn Sie Ihre Einwilligung zu diesen Datenverarbeitungen nicht erklären wollen, ist eine Teilnahme an PRÄP-GO nicht möglich.

Sie können die erklärte Einwilligung jederzeit ohne Angabe von Gründen schriftlich für die Zukunft widerrufen. Ihre Teilnahme an PRÄP-GO endet dann automatisch bzw. ist nicht mehr möglich.

Ihre Rechte in Bezug auf die Verarbeitung Ihrer Daten

In Bezug auf Ihre Daten stehen Ihnen die folgenden Rechte zu:

- Das Recht auf Auskunft über verarbeitete Daten
  (Art. 15 DS-GVO i. V. m. § 83 SGB X)
- Das Recht auf Berichtigung unrichtiger Daten
  (Art. 16 DS-GVO i. V. m. § 84 SGB X)
- Das Recht auf Löschung Ihrer Daten (Art. 17 DS-GVO i. V. m. § 84 SGB X)
- Das Recht auf Einschränkung der Verarbeitung Ihrer Daten

(Art. 18 DS-GVO i. V. m. § 84 SGB X)
- Das Widerspruchsrecht
(Art. 21 DS-GVO i.V. m. § 84 SGB X).

Sie haben das Recht, eine Beschwerde an die Aufsichtsbehörde zu richten, wenn Sie der Meinung sind, dass Ihre Studiendaten unter Verletzung der geltenden Datenschutzgesetze verwendet werden. Dies betrifft Fragen zur Datenverarbeitung und zur Einhaltung der datenschutzrechtlichen Anforderungen durch Ihre behandelnde Klinik, einen Kooperationspartner oder durch die Charité – Universitätsmedizin Berlin.

Für Berlin ist dies die folgende Aufsichtsbehörde:
Berliner Beauftragte für Datenschutz und Informationsfreiheit
Friedrichstraße 219
Tel.: +493013889-0
E-Mail: mailbox@datenschutz-berlin.de

Für Brandenburg:
Die Landesbeauftragte für den Datenschutz und für das Recht auf Akteneinsicht
Stahnsdorfer Damm 77
14532 Kleinmachnow
Telefon:033203/356-0
E-Mail: Poststelle@LDA.Brandenburg.de

Für Hamburg
Der Hamburgische Beauftragte für Datenschutz und Informationsfreiheit
Ludwig-Erhard-Str 22, 7. OG
20459 Hamburg
Tel.: 040 / 428 54 – 4040
E-Mail: mailbox@datenschutz.hamburg.de

Für Bayern:
Bayerischer Landesbeauftragten für den Datenschutz
Postfach 22 12 19
80502 München
E-Mail: poststelle@datenschutz-bayern.de

Für Schleswig-Holstein:
Unabhängiges Landeszentrum für Datenschutz Schleswig-Holstein
Postfach 71 16
24171 Kiel
E-Mail: mail@datenschutzzentrum.de

ChartéCentrum für Anästhesiologie und Intensivmedizin
Charité - Universitätsmedizin Berlin | D - 10117 Berlin

Klinik für Anästhesiologie mit Schwerpunkt operative Intensivmedizin

Direktorin: Univ.-Prof. Dr. C. Spies

Campus Charité Mitte
Charitéplatz 1 | 10117 Berlin
Interne Geländeadresse: Hochhaus, 6.OG
Tel: +49 30 450 531 012
Fax: +49 30 450 531 911
anaesth@charite.de

Campus Virchow-Klinikum
Augustenburger Platz 1 | 13353 Berlin
Interne Geländeadresse: Mittelallee 3
Tel: +49 30 450 551 102
Fax: +49 30 450 551 909
anaesthesie-virchow-klinikum@charite.de

https://anaesthesieintensivmedizin.charite.de

Einwilligungserklärung PRÄP-GOV1.0 vom 08.03.2020 für Patienten und gesetzl. Vertreter

Zu Zwecken der Mitbehandlung und Datenverarbeitung von Forschungszwecken

Patientenaufkleber

Ggf. gesetzlicher Betreuer: ____________________________

A. Erklärung zur Teilnahme an der Studie zur Besonderen Versorgung „Prähabilitation von älteren Patienten mit Gebrechlichkeitssyndrom vor elektiven Operationen (PRÄP-GO)“

Über die Inhalte und den Zweck des Versorgungsangebots, den Behandlungsablauf sowie die Einbeziehung der Charité – Universitätsmedizin Berlin („CHARITÉ“) wurde ich von der behandelnden Ärztin/ dem behandelnden Arzt aufgeklärt. Außerdem habe ich die „Studieninformation für Patienten und gesetzliche Vertreter PRÄP-GO§ 630a BGB“ erhalten und zur Kenntnis genommen und bin mit den dort genannten Inhalten einverstanden.

Mir ist insbesondere Folgendes bekannt:

Meine Teilnahme ist freiwillig. Die Unterzeichnung dieser Erklärung gilt als Teilnahmebeginn.

Nach Ablauf der Widerrufsfrist bin ich an diese Einwilligungserklärung bis zur Durchführung der Nachuntersuchung in meiner Klinik/meinem Krankenhaus gebunden (spätestens 12 Monate nach der Entlassung aus dem Krankenhaus). Eine vorzeitige Beendigung kann nur aus einem wichtigen Grund erfolgen, z.B. aufgrund eines Umzugs oder einer nachhaltigen Störung des Vertrauensverhältnisses zum Arzt.

Meine Teilnahme endet in jedem Fall automatisch mit Beendigung der besonderen Versorgung PRÄP-GO.

Für die Dauer meiner Teilnahme bin ich an die beteiligten Leistungserbringer zur Durchführung der Leistungen dieser besonderen Versorgung gebunden. Andere Leistungserbringer kann ich nur auf Überweisung in Anspruch nehmen oder wenn ich in einem medizinischen Notfall einen Arzt oder Notfalldienst benötige.

Widerrufsbelehrung
Ich kann meine Teilnahmeerklärung innerhalb von zwei Wochen nach ihrer Abgabe in Textform oder zur Niederschrift bei der CHARITÈ ohne Angabe von Gründen widerrufen. Zur Fristwahrung genügt die rechtzeitige Absendung der Widerrufserklärung an die CHARITÈ. Die Widerrufsfrist beginnt mit dieser Belehrung über mein Widerrufsrecht, frühestens jedoch mit der Abgabe meiner Teilnahmeerklärung.

B. Einwilligung in die Verarbeitung meiner personenbezogenen Daten durch die Charité und die Kooperationspartner:

Sie möchten freiwillig an dem Versorgungsforschungsprojekt „Prähabilitation von älteren Patienten mit Gebrechlichkeitssyndrom vor elektiven Operationen" (PRÄP-GO) teilnehmen, um die wissenschaftliche Auswertung dieser besonderen Versorgungsform zu ermöglichen. Die Teilnahme oder Nicht-Teilnahme an dem Forschungsprojekt PRÄP-GOhat keine Auswirkungen auf Ihre übrige medizinische Betreuung.

Aus Gründen des Datenschutzes und der ärztlichen Schweigepflicht ist für die Auswertung der Sie betreffenden personenbezogenen Daten im Rahmen des Forschungsprojekts Ihre freiwillige Einwilligung erforderlich.

Ich **willige ein**, dass die **Charité – Universitätsmedizin Berlin** (Sitz: Charitéplatz 1, 10117 Berlin, vertreten durch den Vorstandsvorsitzenden) und die Kooperationspartner sämtliche personenbezogene Daten, die im Rahmen der Besonderen Versorgung „Prähabilitation von älteren Patienten mit Gebrechlichkeitssyndrom vor elektiven Operationen" (PRÄP-GO) erhoben werden, wie im Folgenden angekreuzt, verarbeiten darf.

Ich erkläre mich damit einverstanden, dass im Rahmen der besonderen Versorgung PRÄP-GOermittelte Gesundheitsdaten (wie bspw. Alter, Geschlecht, Körpergröße, Gewicht sowie Daten zu meiner physischen und psychischen Gesundheit und Daten zur Gebrechlichkeit sowie Kopien des Narkoseprotokolls, der Intensivstationsverlegungsbriefe, des Entlassbrief und der Entlassmedikation) vom Studienpersonal meiner Krankenakte entnommen werden dürfen. Hierfür und nur für diese Angaben entbinde ich meinen behandelnden Arzt/Hausarzt von seiner ärztlichen Schweigepflicht.

☐ Ja ☐ Nein

Ich willige ein, dass die für die Entscheidungsfindungs-Konferenz relevanten Daten (Angaben zu physischer und psychischer Gesundheit) an die Entscheidungsfindungs-Konferenz übermittelt werden dürfen, und dass das Ergebnis der Entscheidungsfindungs-Konferenz (Setting der Prähabilitation, Ziele der Prähabilitation) an die Charité und von der Charité an den Prähabilitationsdienstleister übermittelt werden darf.

☐ Ja ☐ Nein

Ich stimme zu, dass mich das Studienpersonal persönlich, schriftlich oder telefonisch unter Wahrung des Datenschutzes in den nächsten Monaten (auch für die Visiten zu 3 Monaten und 1 Jahr) kontaktieren darf und dafür Zugriff auf meine o.g. personenbezogenen Daten (Kontaktdaten) erhält.

☐ Ja ☐ Nein

Ich bin weiterhin damit einverstanden, dass mein Hausarzt kontaktiert werden darf, falls ich nicht erreichbar bin, um Behandlungsdaten zu erheben und diese ausschließlich für den Zweck der o.g. Studie zu verwenden. Die Daten werden benötigt, um Zusammenhänge zwischen Begleiterkrankungen und der daran verbundenen Behandlung mit kognitiver Leistungsfähigkeit zu erfassen.

Ja ❑ Nein ❑

Kontaktdaten____

Ich bin weiterhin damit einverstanden, dass mein Angehöriger kontaktiert werden kann, falls ich nicht erreichbar bin, um Behandlungsdaten zu erheben und diese ausschließlich für den Zweck der o.g. Studie zu verwenden. Die Daten werden benötigt, um Zusammenhänge zwischen Begleiterkrankungen und der daran verbundenen Behandlung mit kognitiver Leistungsfähigkeit zu erfassen.

Ja ❑ Nein ❑

Kontaktdaten____

Ich bin weiterhin damit einverstanden, dass die Zentren/Ärzte meiner postoperativen Rehabilitation kontaktiert werden dürfen, um Behandlungsdaten zu erheben und diese ausschließlich für den Zweck der o.g. Studie zu verwenden. Die Daten werden benötigt, um Zusammenhänge zwischen Begleiterkrankungen und der daran verbundenen Behandlung mit kognitiver Leistungsfähigkeit zu erfassen.

Ja ❑ Nein ❑

Ich bin weiterhin damit einverstanden, dass eine Anfrage beim zuständigen Einwohnermeldeamt vorgenommen werden kann, falls ich nicht erreichbar bin und diese Daten ausschließlich für den Zweck der o.g. Studie verwendet werden.

Ja ❑ Nein ❑

Ich bin damit einverstanden, dass meine personenbezogenen Daten für das Forschungsvorhaben PRÄP-GOverwendet werden.

Ja ❑ Nein ❑

Ihre kognitiven Leistungsdaten sollen mit der CANTAB® [Cognitive assessment software] erfasst und in dem CANTAB Connect-System (Cloud- https://www.armor.com/) in pseudonymisierter Form an Cambridge Cognition in dem Vereinigten Königreich übermittelt werden. Das Vereinigte Königreich ist ein sogenanntes Drittland, für das noch kein Angemessenheitsbeschluss nach Art. 45 Abs. 1 und 3 DS-GVO der EU-Kommission über ein der Europäischen Union vergleichbares, angemessenes Datenschutzniveau vorliegt. Es bestehen auch noch keine geeigneten Garantien (bestimmte, behördlich genehmigte Datenschutzregeln) im Sinne der Datenschutz-Grundverordnung. Zwar macht es die Pseudonymisierung schwierig, aus den übermittelten Daten Ihre Identität zu ermitteln. Dennoch kann nicht ausgeschlossen werden, dass im Vereinigten Königreich Behörden, Unternehmen oder Privatpersonen Kenntnis von Ihren personenbezogenen Daten und Ihrer Identität erhalten können, die dies in der Europäischen Union nicht dürften. Gegenwärtig wird die Übermittlung der Daten noch als zulässig eingestuft. Sollte bis zum Dezember 2020 kein Angemessenheitsbeschluss getroffen sein, bedarf es für die Datenübermittlung dann Ihrer Einwilligung.
Stimmen Sie für diesen Fall der Übermittlung zu?

 Ja Nein

Ich bin damit einverstanden, dass meine personenbezogenen Daten zu wissenschaftlichen Forschungszwecken im Bereich kognitive und physische Funktionalität verarbeitet werden.

Ja ❑ Nein ❑

Ich bin mit einer schriftlichen Kontaktaufnahme einverstanden ☐ Ja ☐ Nein

Ich bin mit einer telefonischen Kontaktaufnahme einverstanden ☐ Ja ☐ Nein

Ich bin mit dem Besuch eines Mitarbeiters des Kooperationspartners bei mir zu Hause einverstanden ☐ Ja ☐ Nein

Straße ____ Nr. ___________

PLZ _________ Ort ___________________________

Telefon:__________________________ Mobiltelefon:______________________

Am besten tel. erreichbar: ❑ vormittags ❑ nachmittags ❑ abends ❑ sonstiges________

____

Sobald es der Forschungszweck erlaubt, werden die Informationen, mit denen ein Personenbezug hergestellt werden kann, gesondert gespeichert oder die Daten werden anonymisiert. Die Daten werden nach Studienabschluss 10 Jahre aufbewahrt. Danach werden die personenbezogenen Daten gelöscht, soweit nicht gesetzliche satzungsmäßige oder vertragliche Aufbewahrungspflichten entgegenstehen. Mir ist bekannt, dass meine personenbezogenen Daten zu **wissenschaftlichen Forschungszwecken verarbeitet werden**.
Zur Evaluierung von Forschungsfragen werden die pseudonymisierten Daten verarbeitet und ausgewertet zusätzlich durch folgende Dritte:

- Institut für medizinische Informationsverarbeitung, Biometrie und Epidemiologie, Medizinische Fakultät der Ludwig-Maximilians Universität, München; Vertreter: Univ.- Prof. Dr. Ulrich Mansmann
- Fachgebiet Management im Gesundheitswesen, Fakultät Wirtschaft und Management, Technische Universität Berlin, Vertreter: PD Dr. Alexander Geissler.
- Instituts für Biometrie und Klinische Epidemiologie der Charité - Universitätsmedizin Berlin; Vertreterin: Univ. Prof. Dr. Geraldine Rauch,

Die für die studienbedingte Erhebung personenbezogener Daten Verantwortliche im Sinne der Datenschutz-Grundverordnung ist:
Univ.- Prof. Dr. med. Claudia Spies
Campus, Klinik für Anästhesiologie mit Schwerpunkt operative Medizin (CCM/CVK), Virchow-Klinikum
Augustenburger Platz 1
13353 Berlin
Tel.: +49 30 450 55 11 02.

Mir ist bewusst, dass die Charité – Universitätsmedizin Berlin und Kooperationspartner Angaben zu meiner medizinischen Vorgeschichte sowie Angaben zu medizinischen, psychosozialen und funktionellen Fähigkeiten im Rahmen des Versorgungsforschungsprojekt PRÄP-GOerhebt; diese Daten enthalten insbesondere Angaben über meine Gesundheit **(Gesundheitsdaten)**.

Ich habe die Studieninformation zur Teilnahme an der Besonderen Versorgung PRÄP-GOgelesen und verstanden. Meine Fragen bezüglich des Forschungsprojekts wurden zu meiner Zufriedenheit vollständig beantwortet.

Mir ist bekannt, dass diese Einwilligung **freiwillig** ist, und ich sie gegenüber der Charité – Universitätsmedizin Berlin jederzeit mit Wirkung für die Zukunft **widerrufen** kann, indem ich eine entsprechende Mitteilung gegenüber einem Studienmitarbeiter mache oder an praep-go@charite.de sende. Der schriftliche Widerruf kann gegenüber der oben angegebenen Verantwortlichen erklärt werden. Dabei wird die Rechtmäßigkeit der bis zum Widerruf erfolgten Datenverarbeitung nicht berührt. Mir ist bewusst, dass ich meine Daten nicht zu Forschungszwecken zur Verfügung stellen muss und trotzdem an der Besonderen Versorgung PRÄP-GOteilnehmen kann. Die Teilnahme am Forschungsprojekt kann ich jederzeit ohne Angabe von Gründen abbrechen.

C. Hiermit erkläre ich meine Teilnahme und willige in die Datenverarbeitung ein:

(Ort, Datum – Patient/Betreuer)		(Unterschrift Patient/Betreuer)

Vom aufklärenden Arzt auszufüllen:

Der Patient wurde von mir über Ziel, Dauer, Ablauf, Nutzen und Risiken der wissenschaftlichen Untersuchungen im Rahmen der besonderen Versorgungsform mündlich und schriftlich aufgeklärt. Aufgetretene Fragen wurden von mir verständlich und ausreichend beantwortet. Der Patient hat ohne Zwang seine Einwilligung erteilt. Eine Kopie der schriftlichen Studieninformation für Patienten und gesetzl. Vertreter zur Datenverarbeitung im Rahmen der besonderen Versorgung PRÄP-GOin der Version 1.0 und dieser Einwilligungserklärung in der Version 1.0 habe ich dem Teilnehmer ausgehändigt.

(Ort, Datum - vom aufklärenden Arzt einzutragen)		(Unterschrift aufklärender Arzt – Vor- und Nachname)

Name/Stempel aufklärender Arzt in Druckbuchstaben

Einwilligung in die Übermittlung personenbezogener Daten durch [Prähabilitationsdienstleister xxx] an die Charité

Zusätzlich willige ich darin ein, dass [Prähabilitationsdienstleister xxx], die mich betreffenden im Rahmen der Besonderen Versorgung PRÄP-GOerhobenen Daten an die Charité zum Zweck der **wissenschaftlichen Auswertung** übermitteln darf. Diese Daten enthalten Angaben über meine Gesundheit (**Gesundheitsdaten**).

Diese Einwilligung ist **freiwillig** und kann ebenfalls jederzeit ganz oder teilweise durch entsprechende Mitteilung an [email@beispiel.de] oder schriftlich **widerrufen** werden. Durch den Widerruf der Einwilligung wird die Rechtmäßigkeit der aufgrund der Einwilligung bis zum Widerruf erfolgten Verarbeitung nicht berührt.

Den schriftlichen Widerruf der Einwilligung in die Übermittlung personenbezogener Daten an die Charité richten Sie bitte an:

[Name des Prähabilitationsdienstleister]

[Adresse]

______________________________	____
Ort und Datum	Unterschrift Patient/in oder Betreuer

Einwilligung in die Übermittlung personenbezogener Daten durch [Krankenkasse xxx] an die Charité

Zusätzlich willige ich darin ein, dass [Krankenkasse xxx] die mich betreffenden Daten zu Leistungsausgaben (hierzu gehören die Kostendaten zu ärztlichen und zahnärztlichen Behandlungen, zu Arznei-, Heil- und Hilfsmitteln, zu Krankhausbehandlungen sowie zu Rehabilitationsleistungen, aber auch alle anderen Leistungsausgaben) an die Charité – Universitätsmedizin Berlin zum Zweck der **wissenschaftlichen Auswertung** der besonderen Versorgungsform übermittelt. Diese Daten enthalten Angaben über meine Gesundheit (**Gesundheitsdaten**).

Diese Einwilligung ist **freiwillig** und kann ebenfalls jederzeit ganz oder teilweise durch entsprechende Mitteilung an [email@beispiel.de] oder schriftlich **widerrufen** werden. Durch den Widerruf der Einwilligung wird die Rechtmäßigkeit der aufgrund der Einwilligung bis zum Widerruf erfolgten Verarbeitung nicht berührt.

Den schriftlichen Widerruf der Einwilligung in die Übermittlung personenbezogener Daten an die Charité richten Sie bitte an:

[Name der Krankenkasse]

[Adresse]

______________________________	____
Ort und Datum	Unterschrift Patient/in oder Betreuer
