# Supplementary material for: Prehabilitation of elderly frail or pre-frail patients prior to elective surgery (PRAEP-GO): study protocol for a randomized, controlled, outcome assessor-blinded trial
Source: Trials. 2022 Jun 6;23:468. doi: 10.1186/s13063-022-06401-x (PMC9167908; doi:10.1186/s13063-022-06401-x)
Supplement: Supplementary file 4 — Additional file 4. Spirit Figure. [file 13063_2022_6401_MOESM4_ESM.pdf]

Additional File 1: assessments at all visits

**ASSESSMENTVERFAHREN**		**STUDY PHASE**																	
		**I**		**II**	**III**		**IV**												
**Category**	**Measured data**	**V0**	**V2**	**V3**	**V4**	**V5**	**V6**	**V7**	**V8**	**V9**	**V10**	**V11**	**V12**	**V13**	**V14**	**V15**	**V16**	**V17**	**V18**
**Sociodemographic Data**	Height	X																	
	Weight	X				X	X			X									X
	BMI	X				X	X			X									X
	Sex	X																	
	Smoking (FAGERSTROM)		X							X									X
	Alcohol consumption (AUDIT-C)		X							X									X
	Life situation		X																
**Anamnesis**	Daily medication		X				X			X			X			X			X
	Pain medication		X				X			X			X			X			X
	Charlson Comorbidity Index (CCI)		X							X									X

ASSESSMENTVERFAHREN		STUDY PHASE																	
		I		II	III		IV												
**Anämie**	Blood Count (from Routine)		X																
**Muscle strength**	Handgrip strength	X				X				X									X
**Endurance**	2-minute-step-test (2-M-ST)		X			X				X									X
	Expiratory Peak-Flow		X							X									X
**Mobility**	Timed Up&Go (TUG)		X			X				X									X
	CHARMI		X				X			X									X
	Gait speed over 15 ft.	X				X	X			X									X
	Metabolic Equivalents (MET)		X			X				X									X
	Stair climbing speed (SCS)		X		X	X				X									X
	Subjective fatigue	X				X	X			X									X
	Life-Space-Assessment (LSA)		X							X									X

ASSESSMENTVERFAHREN		STUDY PHASE																	
		I		II	III		IV												
**Pain**	Numeric Rating Scale (NRS)		X			X	X	X	X	X	X	X	X	X	X	X	X	X	X
**Nutrition**	Weight Loss	X				X	X			X									X
	Albumin		X				X												X
	Mini Nutritional Assessment, SF		X							X									X
	Calf circumference		X							X									X
	Upper Arm circumference		X							X									X
**Cognition**	Mini Cog	X								X									X
	MOCA		X							X									X
	CANTAB Test battery		X							X									X
	Multifactorial Memory Questionnaire (MMQ)		X							X									X
	Trail making Test A and B		X							X									X

ASSESSMENTVERFAHREN		STUDY PHASE																	
		I		II	III		IV												
	Informant Questionnaire on Cognitive Decline in the Elderly (IQCODE)		X							X									X
	Screening / diagnosis of Neurocognitive Disorders		X							X									X
**Fall risk**	Falls in the past year		X																
	Fall incidence					X	X	X	X	X	X	X	X	X	X	X	X	X	X
	ABC-6-Scale		X			X				X									X
**Quality of Life**	Quality of Life (EQ-5D-5L)		X			X				X			X			X			X
	Anxiety (GAD-7)		X							X									X
	Subj. Health (PHQ-8)		X							X									X
	CarerQol			X						X									X
**Subjective Health**	WHODAS 2.0, 12 Items		X							X									X
**Evaluation of the Intervention**	Expectation for pending Intervention		X	X		X													

ASSESSMENTVERFAHREN		STUDY PHASE																	
		I		II	III		IV												
	Satisfaction with Intervention (ZUF-8)					X													X
	Satisfaction with process							X											X
**Social**	Activities of Daily Living (ADL/iADL)		X							X			X			X			X
	Social Situation according to Nikolaus (SOS), Subscale 1 and 2		X							X									X
	Loneliness (3-Item-Loneliness Scale)		X							X									
**Perception**	Hearing Impairment		X							X									X
	Seeing impairment		X							X									X
**Shared-Decision-Making**	Autonomy Preference Index (API)			X															
	9-item Shared Decision Making Questionnaire (SDM-Q-9), medical doctor				X														

ASSESSMENTVERFAHREN		STUDY PHASE																	
		I		II	III		IV												
	SDM-Q-9, Patient				X														
	SDM-Q-9, informal carer				X														
**Prehabilitation**	Defined goals (primary, secondary) for prehabilitation			X															
	Performed therapies during prehabilitation					X													
	Patient diary für independent exercises					X													
**Care dependency**	Level of care dependency (NBA)		X							X									X
	Current level of care dependency		X							X									X
	Barthel Index (BI)		X				X			X									X
**Health Care Utilization**	Visits medical doctor / hospital							X		X			X			X			X
	LOS hospital							X		X			X			X			X

ASSESSMENTVERFAHREN		STUDY PHASE																	
		I		II	III		IV												
	Onset of new medical conditions							X		X			X			X			X
	Mortality							X											X
	FIMA		X							X			X			X			X
**Documentation in patient file**	ASA-Score		X																
	Surgical risk, Type		X				X												
	Duration of surgery and anaesthesy						X												
	Level of severity of surgical procedure (Possum-Score)						X												
	Anesthesiology procedure						X												
	Duration ICU (if any)						X												
	Surgical procedure (OPS-Code)						X												

ASSESSMENTVERFAHREN		STUDY PHASE																	
		I		II	III		IV												
	LOS in hospital after surgical procedure						X												
	Complications during Hospital stay						X												
	Time until first mobilization						X												
	Time until Standing						X												
	Time until walking (without help)						X												
	Patient diary in hospital						X												
	Duration of treatment in recovery room or ICU						X												
**Ergänzende Angaben**	Reason for transferal on ICU, Intervention						X												
	Intensive Care scores (SAPS II, SOFA, APACHE II, TISS-28)	Inte ns					X												

ASSESSMENTVERFAHREN		STUDY PHASE																	
		I		II	III		IV												
	Beatmungs- einstellungen und -dauer der invasiven- und nicht invasiven Beatmung						X												
	Routinelabor						X												
	concomitant medication during hospital stay						X												
	Analgesic needs during surgical procedure (pre-, peri, post-surgical)						X												
	Survival						X	X	X	X	X	X	X	X	X	X	X	X	X
