# Supplementary material for: Prehabilitation of elderly frail or pre-frail patients prior to elective surgery (PRAEP-GO): study protocol for a randomized, controlled, outcome assessor-blinded trial
Source: Trials. 2022 Jun 6;23:468. doi: 10.1186/s13063-022-06401-x (PMC9167908; doi:10.1186/s13063-022-06401-x)
Supplement: Supplementary file 5 — Additional file 5. Study protocol. [file 13063_2022_6401_MOESM5_ESM.pdf]

Study Protocol

Prehabilitation of elderly patients with frailty syndrome prior to elective surgery - Randomised controlled multicentre study

(PRÄP-GO)

**German study title:**	Prähabilitation von älteren Patienten mit Gebrechlichkeitssyndrom vor elektiven Operationen – randomisiert kontrollierte multizentrische Studie
**Principal Investigator:**	Univ.-Prof. Dr. Claudia Spies<br>Email: claudia.spies@charite.de
**Coordinating centre:**	Department of Anesthesiology<br>and Operative Intensive Care Medicine<br>Charité - Universitätsmedizin Berlin<br>**Campus Charité Mitte**<br>Charitéplatz 1, 10117 Berlin<br>Internal address: Hochhaus, 6.OG<br>Tel: +49 30 450 531 012<br>Fax: +49 30 450 531 911<br>anaesth@charite.de<br><br>**Campus Virchow-Klinikum**<br>Augustenburger Platz 1 | 13353 Berlin<br>Internal address: Mittelallee 3, 2.OG<br>Tel: +49 30 450 551 102<br>Fax: +49 30 450 551 909<br>anesthesie-virchow-klinikum@charite.de<br>https://anaesthesieintensivmedizin.charite.de
**Protocol UTN No.:**	U1111-1253-4820
**Clinical Trials:**	NCT04418271
**Protocol Version:**	Version 1.1 of 13.06.2020
**PRÄP-GO Website:**	www.praep-go.de

Financing: The new form of care is funded by the Innovation Committee of the Federal Joint Committee based in Berlin (grant number 01NVF18024).

Contents

1 List of Abbreviations....5
2 Synopsis....7
2.1 German Synopsis....7
2.2 English Synopsis:....9
3 Administrative structures....12
3.1 Coordinating centre and study management centre....12
3.2 Management committee....12
3.3 Data and Safety Monitoring Committee (DSMC)....12
4 Preface....14
5 Lines of Inquiry....15
5.1 Primary question....15
5.2 Secondary questions....15
6 Study Endpoints....17
6.1 Primary endpoint....17
6.2 Secondary endpoints....17
6.2.1 Other secondary goals....18
6.2.2 Final goals in the intervention group....18
6.2.3 Influencing parameters....18
7 Study Structure....19
7.1 Study design....19
7.2 Study population....19
7.3 Inclusion and exclusion criteria....19
7.3.1 Inclusion criteria of the study group....19
7.3.2 Exclusion criteria of the study group....19
7.3.3 Descriptive controls (optional)....20
7.3.4 Family members....20
7.3.5 NCD control group (optional)....20
7.4 Inclusion....20

7.5 Recruitment ... 20
7.6 Course of the study ... 21
7.7 Investigations and assessments ... **Fehler! Textmarke nicht definiert.**
7.7.1 Organisation of visits ... 23
7.7.2 Classification of assessment procedures in the domains of the ICF ... 23
7.7.3 Evidence of the assessment procedures used ... 24
7.8 Randomisation ... 24
7.9 Intervention ... 25
7.9.1 General description ... 25
7.9.2 Shared Decision Making (SDM) conference ... 25
7.9.3 Prehabilitation ... 26
7.9.4 Training plan ... 28
7.9.5 Training schedule ... 29
7.9.6 Details of prehabilitation services and therapy ... 29
7.10 Follow-up ... 30
7.10.1 Description ... 30
7.10.2 Visit 6 ... 30
7.10.3 Visits 7, 8, 10 - 17 ... 30
7.10.4 Visit 9 ... 31
7.10.5 Visits 12 and 15 ... 32
7.10.6 Visit 18 ... 32
8 Ethics ... 33
9 Data Management ... 34
9.1 Collection methods ... 34
9.2 Data management ... 34
9.3 Data quality and monitoring ... 34
9.4 Protocol deviations ... 34
10 Statistics ... 36
10.1 Power calculation and sample size ... 36

10.2 Analysis of the results 36
10.2.1 Primary endpoint 37
10.2.2 Secondary endpoints 37
11 Safety 39
11.1 Adverse events 39
11.2 Safety 39
11.3 Reporting 39
12 Other 40
12.1 Financing of the study 40
12.2 Publication guidelines 40
12.3 Study Registration 40
13 Literature/References 41
Annexes 44

List of Tables

Table 1: Phases and visits in the planned evaluation of PRÄP-GO 23
Table 2: Classification of assessment procedures in the domains of the ICF 24
Table 4: Intervention forms and therapy goals 27
Table 5: Training plan and focus shown by week in the prehabilitation program 29
Table 6: Data collected during telephone follow-up surveys 31
Table 7: Quantification of the intervention effect based on Müller-Mai et al. (2015) 36

List of Figures

Fig. 1: Course of study 22
Fig. 2: Application of the three-talk model in the PRÄP-GO project 25

1 List of Abbreviations

2-MST:	2-minute step test
ABC:	Activities and Balance Confidence Scale
AHB:	Follow-up Treatment (Anschlussheilbehandlung)
APACHE II:	Acute Physiology, Age, Chronic Health Evaluation
API:	Autonomy Preference Index
ASA:	American Society of Anesthesiologists classification
AUDIT:	Alcohol Use Disorders Identification Test
BI:	Barthel Index
BMI:	Body Mass Index
BSSS:	Berlin Social Support Scale
CCI:	Charlson Comorbidity Index
CHARMI:	Charité Mobility Index
CRP:	C-reactive protein
EQ-5D-5L:	EuroQol quality of life questionnaire
ET:	Ergotherapy
FIMA:	Questionnaire on the utilisation of medical and non-medical services for the elderly
GAD:	Generalised Anxiety Disorder Scale
GDS:	Geriatric Depression Scale
GGT:	Gamma Glutamyl Transferase
GOLD:	Global Initiative for Chronic Obstructive Lung Disease
GPT:	Glutamate Pyruvate Transaminase
INR:	International Normalised Ratio
ICU:	Intensive Care Unit
KH:	hospital (Krankenhaus)
LSA:	Life Space Assessment
MdK:	Medical service of the health insurance companies
MET:	Metabolic equivalent of tasks
MNA:	Mini Nutritional Assessment
MOCA:	Montreal Cognitive Assessment

NBA:	New Nursing Care Grading Assessment (Neues Begutachtungsassessment)
NRS:	Numerical Rating Scale
NYHA:	New York Heart Association Scale
OP:	Surgery
OPS:	Operation and procedure identification key
PEF:	Participatory Decision Making
PHQ:	Patient Health Questionnaire
PT:	Physiotherapy
PTT:	Partial thromboplastin time
RCT:	Randomised Controlled Trial
SAPS II:	Simplified Acute Physiology Score
SDM:	Shared Decision Making
SOFA:	Sepsis-related Organ Failure Assessment score
SOS:	Social Situation questionnaire by Nikolaus
TU:	Therapy units
TUG:	Timed Up & Go test
TISS-28:	Therapeutic Intervention Scoring System
TSG:	Stair climbing speed (Treppensteigegeschwindigkeit)
TTM	Three-Talks Model
UCLA:	University of California, Los Angeles
WHODAS:	World Health Organisation Disability Assessment Schedule
ZUF:	Patient Satisfaction Questionnaire
ZZ:	meantime (Zwischenzeit)

2 Synopsis

2.1 Synopsis (German)

**Hintergrund**	Aufgrund der steigenden Lebenserwartung der Bevölkerung und aufgrund des medizinischen Fortschritts erfolgen mehr und komplexere chirurgische Eingriffe bei älteren Patienten. Auch wenn oftmals der direkte postoperative Verlauf aus medizinischer Sicht komplikationsfrei verläuft, zeigen viele Patienten mittel- und langfristige Einschränkungen in Hinblick auf die Patientenautonomie, kognitive und funktionelle Fertigkeiten und weitere Domänen der Lebensqualität (Saczynski et al. 2012). Verschiedene Studien zeigen, dass ein Gebrechlichkeitssyndrom (englisch: Frailty-Syndrome) einen Risikofaktor für ein schlechtes postoperatives Outcome darstellt (Watt et al. 2018; Birkelbach et al. 2019).<br><br>Dabei stellt ein Gebrechlichkeitssyndrom jedoch keine Diagnose eines festen Zustands dar, sondern eher eine Beschreibung eines fluiden, sich verändernden Zustands (Lorenzo-López et al. 2017) . Aus diesem Grund lässt sich dieser Zustand auch mittels gezielter Interventionen positiv beeinflussen (de Labra et al. 2015). In der Vorbereitung auf eine anstehende Operation geschieht dies durch eine so genannte Prähabilitation. Dabei handelt es sich um eine zielgerichtete multimodale Therapie zur Behandlung bestehender Symptome des Frailty-Syndroms vor der Operationsdurchführung (Gurlit and Gogol 2019). Die Prähabilitation reduziert perioperative Komplikationen (Lin et al. 2016), unterstützt die postoperative funktionelle Wiederherstellung (Gill et al. 2004; Swank et al. 2011) und verringert die Gefahr von postoperativen kognitiven Störungen wie zum Beispiel dem postoperativen Delir (Deschodt et al., 2012; Friedman et al., 2009; Gustafson et al., 1991; Moyce et al., 2014). Verschiedene Studien konnten zudem eine kürzere postoperative Verweildauer im Krankenhaus nachweisen (Lin et al. 2016; Chen et al. 2018).<br><br>Aus diesem Grund werden im Projekt PRÄP-GO Patienten mit einem Gebrechlichkeitssyndrom im präoperativen Umfeld identifiziert und vor der Durchführung der geplanten Operation einer Prähabilitation zugeführt., In einem multidisziplinären Setting werden vorhandene Risikofaktoren für perioperative Komplikationen verringert oder

	beseitigt. Zu den in diesem Setting stattfindenden Maßnahmen gehören unter anderem trainingstherapeutische Maßnahmen, Alltagstraining oder eine Ernährungsberatung, aber auch die Begutachtung des aktuellen Medikamentenplans mit dem Ziel der Vermeidung einer Polypharmazie. Zur Stärkung der Patientenpartizipation wird die Auswahl von Behandlungszielen und Maßnahmen der Prähabilitation in einer gemeinsamen Entscheidungsfindungskonferenz (Shared Decision Making Konferenz) mit den behandelnden Ärzten, Pflegekräften/Physiotherapeuten und dem Patienten stattfinden. Die neue Versorgungsform der Prähabilitation soll innerhalb des Projekts PRÄP-GO evaluiert werden. Dabei stehen die Beurteilung der Effektivität der Intervention, d.h. der Verringerung der postoperativen Pflegeabhängigkeit 1 Jahr nach OP sowie die gesundheitsökonomische Betrachtung im Mittelpunkt.
**Ziel**	Primäres Ziel ist der Nachweis, dass eine dreiwöchige Prähabilitation in Patienten mit Gebrechlichkeitssyndrom ≥ 70 Jahre alt, bei denen eine elektive Operation geplant ist, zur Verminderung der Pflegeabhängigkeit ein Jahr postoperativ führt.<br><br>Eine gesundheitsökonomische Analyse wird ebenfalls durchgeführt.
**Design**	Multizentrische, nationale, randomisierte, kontrollierte, Outcome-Assessor verblindete pragmatische klinische Studie mit 1400 Patienten. Die Randomisierung erfolgt als zweiarmiges paralleles Gruppendesign, Allocation ratio 1:1 pro Krankenhaus.
**Methode**	Patienten werden eingeschlossen, wenn sie alle Einschluss-, aber kein Ausschlusskriterium erfüllen.<br><br><u>Einschlusskriterien:</u><br>- Alter ≥ 70 Jahre<br>- Einwilligungsfähiger Patient oder vorhandener gesetzlicher Betreuer bei nicht-einwilligungsfähigem Patient<br>- Elektive Operation geplant<br>- Erwartete Anästhesiedauer ≥60 min<br>- Versicherung in der gesetzlichen Krankenversicherung*<br>- Frailty-Syndrom (≥1 positiver von 5 standardisierten Parametern entsprechend dem Physical Frailty Phänotyp nach Fried et. al. (Fried et al., 2001)

	* Rechtsgrundlage: Selektivertrag gem. §140a SGB V; alternative Rechtsgrundlage nach Freigabe durch die Projektleitung und Projektträger Behandlungsvertrag gem. §630a BGB, wenn kein Selektivvertrag vorhanden.<br><br><u>Ausschlusskriterien:</u><br>- Schwere kardiologische oder pulmonale Erkrankung (NYHA IV, Gold IV)<br>- Intrakranielle Eingriffe<br>- Moribunde Patienten (palliative Situation)<br>- Keine ausreichenden Sprachkenntnisse<br>- Teilnahme an einer anderen interventionellen Rehabilitationsstudie oder einer AMG- bzw. MPG-Studie, die nicht von der Projektleitung freigegeben wurden (Ausnahme: Parallele Teilnahme an adjuvanter Therapiestudie).<br>- Keine rechtsgültige Einwilligungserklärung
**Outcome**	**Primärer Endpunkt:** Die Veränderung der Pflegestufe ein Jahr postoperativ. Die Festlegung der Pflegestufe erfolgt mittels gewichteten Punktwert des Neuen Begutachtungsassessments (NBA) (Wingenfeld et al., 2008).<br><br>**Wesentliche Sekundäre Endpunkte** sind:<br>- Frailty-Entwicklung bis 12 Monate postoperativ<br>- Lebensqualität nach 12 Monaten postoperativ<br>- Mortalität<br>- Gesundheitsökonomische Parameter<br>- Evaluation der Entscheidungsfindungskonferenz
**Studienregistrierung**	ClinicalTrials.gov (NCT04418271)<br><br>Universal Trial Number (UTN): U1111-1253-4820

2.2 Synopsis (English):

**Background**	Due to an increase in the life-expectancy of the general population and continuous advancements in the medical field, the number and complexity of surgical interventions in elderly patients has been growing steadily for years. Although the direct postsurgical process is often considered a success, middle- and long-term observations suggest that many of these patients lose some degree of autonomy, cognitive and functional capacity, and suffer a marked decrease in other domains of quality of life (QoL) (Saczynski et al. 2012). With increasing age and a subsequent reduction in physiological reserves, a

	state of vulnerability emerges – the frailty syndrome – involving limitations in functional reserves, mobility, muscle strength, and vital capacity. Frailty syndrome has been shown to be a significant risk factor for postoperative complications and mortality (Watt et al. 2018; Birkelbach et al. 2019).<br><br>Frailty, however, is not a permanent diagnosis. Instead, it describes a fluid, and often reversible, state of condition for a given patient (Lorenzo-López et al. 2017). Frailty status can be positively influenced by specific interventions (de Labra et al. 2015). Such interventions may be employed in preparation for a planned surgical procedure, in terms of a so-called prehabilitation program, which includes a specific, multimodal therapeutic concept for the treatment of frailty aspects prior to surgery (Gurlit and Gogol 2019). Prehabilitation is associated with a reduction in perioperative complications (Lin et al. 2016), such as delirium (Deschodt et al., 2012; Friedman et al., 2009; Gustafson et al., 1991; Moyce et al., 2014), and enhanced functional recovery following surgery (Gill et al. 2004; Swank et al. 2011). Additionally, patients and health systems alike can profit from a shortened length of stay following surgery (Chen et al. 2018).<br><br>The objective of PRÄP-GO is to establish and employ a suitable preoperative case-care management system to improve the short and long-term outcome of elderly surgical patients with signs of a frailty syndrome, improving postoperative quality of life and reducing institutionalisation rates using a three-week individualised prehabilitation program.
**Aim**	The aim of the study is to evaluate the effect of a shared decision making conference and three week prehabilitation program on the outcome "care dependency" one year after surgery. The cost-effectiveness of the intervention will also be evaluated.
**Design**	A 1400 patient, national multicentre, assessor-blinded, randomised, pragmatic, controlled, parallel-group, clinical trial. Randomisation is planned as a two-armed, parallel group design with a 1: 1 allocation ratio for each participating hospital.

**Method**	Patients are included if they meet all inclusion criteria and no exclusion criteria.<br><br>**Inclusion criteria:**<br>- Age ≥ 70 years<br>- Patient able to provide consent or existing legal guardian in case of patient is unable to provide consent<br>- Planned elective surgery<br>- Expected anaesthesia duration ≥60 min<br>- Insured through the statutory health insurance*<br>- Frailty Syndrome (≥1 positive parameters of 5 standardised parameters corresponding to Fried's Physical Frailty Phenotype (Fried et al., 2001)<br><br>* Legal basis is a selective contract according to §140a SGB V with the insurance company; an alternative legal basis, after approval by the project management and project funding agency, is a treatment contract according to §630a BGB if no selective contract exists.<br><br>**Exclusion criteria:**<br>- Severe cardiological or pulmonary disease (NYHA IV, Gold IV)<br>- Intracranial procedures<br>- Moribund patients (palliative situation)<br>- Language barrier<br>- Participation in another interventional rehabilitation study or an AMG or MPG study that has not been approved by the project management (exception: parallel participation in adjuvant therapy studies).<br>- No legally valid declaration of consent.
**Outcome**	**Primary endpoint:** Change in care dependency level one year postoperatively. The care dependency level is determined by means of the weighted point value of the new nursing care grading assessment (NBA) (Wingenfeld et al., 2008).<br><br>Key secondary endpoints are:<br>- Frailty status up to 12 months postoperatively<br>- Quality of Life after 12 months postoperatively<br>- Mortality<br>- Health economic parameters<br>- Evaluation of the shared-decision making conference
**Registration**	ClinicalTrials.gov (NCT NCT04418271)<br><br>Universal Trial Number (UTN): U1111-1253-4820

3 Administrative structures

3.1 Coordinating centre and study management centre

Department of Anesthesiology and Operative Intensive Care Medicine
Charité – Universitätsmedizin Berlin

Campus Charité Mitte
Charitéplatz 1 | 10117 Berlin (Germany)
Internal address: Hochhaus, 6th floor

Campus Virchow-Klinikum
Augustenburger Platz 1 | 13353 Berlin (Germany)
Internal address: Mittelallee 3, 2nd floor

E-mail: project-praep-go@charite.de
Telephone: +49 (0)30 - 450 63 12 45
Webpage: www.praep-go.de

3.2 Management committee

Title	Name	First name	Main responsibility in the project
Univ.-Prof. Dr.	Claudia	Spies	Principal Investigator/Project Director
Univ.-Prof. Dr.	Stefan	Schaller	Deputy Project Director
Dr.	Katrin	Schmidt	Project Manager
Dr.	Jörn	Kiselev	Supervisor of Therapeutic Prehabilitation
	Rudolf	Mörgeli	Supervisor Frailty
Univ.-Prof. Dr.	Ulrich	Mansmann	Project Evaluation

3.3 Data and Safety Monitoring Committee (DSMC)

An independent Data and Safety Monitoring Committee (DSMC) consisting of experts in clinical studies, biostatistics and perioperative medicine is to be established to monitor recruitment, protocol adherence, as well as monitoring follow-up and safety data.

Virtual meetings and a face-to-face meeting at LIVES 2021 are planned. The project management as well as the DSMC may independently request video conferences.

The results of all meetings are logged. Detailed information on the tasks and members of the DSMC and regulations on the meetings are available in the Annex 1 - DSMC regulations.

Management committee signature page

The Management Committee has read the PRÄP-GO protocol version 1.1 dated 13th of June, 2020 and approves it as the official protocol for the study - Prehabilitation of elderly patients with frailty syndrome prior to elective surgery - Randomised controlled multicentre study (PRÄP-GO).

Claudia Spies (Principal Investigator)		Date:
Stefan Schaller		Date:
Katrin Schmidt		Date:
Jörn Kiselev		Date:
Rudolf Mörgeli		Date:
Ullrich Mansmann		Date:

4 Preface

Due to the increasing life expectancy of the population and significant medical progress, more and more complex surgical interventions are being carried out in older patients. This goes hand in hand with higher demands on the indication for surgery, patient safety and the quality of postoperative care. Although the direct postoperative phase is often without complications from a medical perspective, many patients show medium and long-term restrictions with regard to patient autonomy, cognitive and functional skills, as well as other domains of quality of life (Saczynski et al. 2012). Much like diabetes mellitus (Martin et al. 2016; Sibia et al. 2018) or cardiovascular disease (Smilowitz et al. 2018), a frailty syndrome is an independent risk factor for the occurrence of postoperative complications (Watt et al. 2018; Birkelbach et al. 2019), and is associated with a longer hospitalisation periods, deterioration of functional capacities and quality of life (Sepehri et al. 2014; Lin et al. 2016).

Regardless of a surgery intervention, frailty syndrome is associated with restrictions in the functional reserve, including mobility, muscle strength and vital capacity. It is an independent risk factor for the development of postoperative complications such as postoperative delirium (Leung, Tsai, and Sands 2011), infections, congestive heart failure, immobility and long-term care (Kim et al. 2014) or long-term cognitive disorders (Rana, Bonasera, and Bordelon 2017). The prevalence of frailty syndrome is estimated to be between 4.0% and 27.3% of the population ≥ 65 years (Santos-Eggimann et al. 2009). In the perioperative setting, the prevalence is up to 50%, depending on the surgical discipline (Moyce, Rodseth, and Biccard 2014a; Sepehri et al. 2014). Patients with frailty syndrome benefit from prehabilitation, i.e. targeted treatment of individual components of frailty syndrome. Various studies show a faster post-operative recovery of functional skills (Gillis et al. 2018; Jahic et al. 2018), reduction of postoperative complications, such as cognitive disorders or a postoperative delirium (Moyce, Rodseth, and Biccard 2014b; Deschodt et al. 2012; Gustafson et al. 1991; Friedman et al. 2009), and shorter hospital stays (Chen et al. 2018). However, most of these findings only apply to certain patient groups and / or types of surgery, and so far there have been no corresponding studies for generalizable evidence. The identification of patients with frailty syndrome is possible as part of the preoperative routine (Birkelbach et al. 2017).

For this reason, a clinical system for the identification of patients with frailty or pre-frailty in the preoperative environment is to be established in the PRÄP-GO project. Identified patients are to undergo a prehabilitation program prior to the operation . Here, existing risk factors are discussed in a multidisciplinary setting, along with strategies to reduced or eliminated perioperative complications. Such strategies include not only training measures, training of daily activities and nutritional counselling, but also a review of the current medication plan with the aim of reducing daily intake and to avoid undesirable side effects.

This system is to be evaluated within the PRÄP-GO project. The focus is on the effectiveness of the intervention (in terms of preventing postoperative nursing care dependency one year after surgery) and the health economic considerations.

5 Lines of Inquiry

5.1 Primary question:

Can a prehabilitation program prior to elective surgery in the elderly, aiming to reduce risk factors for perioperative complications due to a frailty syndrome, prevent nursing care dependency levels from worsening one year after surgery?

5.2 Secondary questions:

1. Is such a prehabilitation suitable to maintain or improve the functional capacities of the study participants?
    a. Immediatela after the intervention?
    b. Does prehabilitation have an impact on functional status 12 months after undergoing elective surgery?
2. What is the effect of prehabilitation on the following parameters within 12 months after the operation:
    a. Fear of falling
    b. Quality of life
    c. Length of stay
    d. Number of transfers to ICU
    e. Number of postoperative complications
    f. Course of rehabilitation / follow-up treatment
    g. Number of doctor visits in the follow-up period
    h. Number and length of hospitalisations in the follow-up
    i. Change in accessibility of living spaces
3. Does a positive expectation of the effectiveness of a prehabilitation influence the adherence during the prehabilitation?
4. Is a higher adherence to prehabilitation associated with an improved effectiveness?
5. Socio-economic factors and consequences
6. Health economic evaluation: is prehabilitation cost-effective?
    a. What does prehabilitation cost?
    b. What is the cost difference between the intervention group and the control group from the time of inclusion to the end of the follow-up?
    c. What is the number of quality-adjusted years of life (QALYs) of the intervention group and control group over the follow-up period?
    d. What is the difference between costs for the intervention and control groups?
    e. Are there differences among benefit parameters (QALYs, level of nursing care dependency, mortality, WHO DAS) for the intervention and control groups?

   f. What is the relationship between costs and benefits, i.e. what are the costs per additional QALY? And is this relation in an acceptable range?
7. What are the requirements to ensure that prehabilitation as a new health care feature is successfully implemented in the German health care system?

6 Study Endpoints

6.1 Primary endpoint

Level of nursing care dependency (according to the new nursing care grading assessment (NBA)) (Wingenfeld et al., 2008) one year after surgery

6.2 Secondary endpoints

1. Cognition
    - Incidence of mild or major neurocognitive disorder (NCD)
      Measurement duration: preoperatively, 3 and 12 months postoperatively
    - Dementia risk (MOCA)
      Measurement duration: up to 1 year postoperatively
    - Incidence of cognitive impairment
      Measurement duration: up to 1 year postoperatively
2. Degree of frailty (Fried's Physical Phenotype)
   Measurement duration: up to 1 year postoperatively
3. Intraoperative neuromonitoring (power spectrum alpha band, spectral corner frequency and burst suppression)
   Measuring time: During the operation
4. Operation outcome based on complications rates (intraoperative and postoperative, categories: no complications, with complications including hypothermia, hypotension, bleeding, infections, re-interventions)
   Measurement duration: up to 1 year postoperatively
5. Extent of patient participation preference: autonomy preference index, German modified version (API-Dm) and questionnaire for participatory decision making (PEF-FB-9))
   Measuring time: Prior to prehabilitation start
6. Nutritional status MNA-SF
   Measurement duration: up to 1 year postoperatively
7. Sarcopenia, calf circumference, arm circumference
   Measurement duration: up to 1 year postoperatively
8. Mobility and functional status
    - Change in mobility (TUG, ability to climb stairs, walking speed)
      Measurement duration: up to 1 year postoperatively
    - Change in social mobility, measured with the LSA
      Measurement duration: up to 1 year postoperatively
    - Change in functional mobility in the direct postoperative course (CHARMI)
      Measuring time: During the hospital stay
9. Need for medicinal substances and assistive equipment
   Measurement duration: up to 1 year postoperatively
10. Depression and anxiety (PHQ 8, GAD 7)
    Measurement duration: up to 1 year postoperatively
11. Quality of Life (EQ-5D-5L)
    Measurement duration: up to 1 year postoperatively
12. Patient-reported function and disability (WHO Disability Assessment Schedule)
    Measurement duration: up to 1 year postoperatively
13. Proxy-reported function and disability (WHO Disability Assessment Schedule)
    Measurement duration: up to 1 year postoperatively
14. Falls (incidence), risk of falling (TUG, walking speed) and fear of falling (ABC-6)
    Measurement duration: up to 1 year postoperatively
15. Social situation (BSSS)
    Measurement duration: up to 1 year postoperatively
16. Pain (NRS)

Measurement duration: up to 1 year postoperatively

17. Satisfaction with the overall process (ZUF-8)
    Measurement duration: up to 1 year postoperatively
18. Loneliness (UCLA loneliness questionnaire)
    Measurement duration: up to 1 year postoperatively
19. Survival
    Measurement duration: up to 1 year postoperatively

6.2.1 Other secondary end goals:

1. Intensive care treatment period
   Measurement duration: During the hospital stay (up to 1 year postoperatively)
2. Intensive care unit admission (planned / unplanned)
   Measurement duration: During the hospital stay (up to 1 year postoperatively)
3. Hospital stay
   Measurement duration: During the hospital stay (up to 1 year postoperatively)
4. Discharge modality (destination following hospital discharge)
   Measurement duration: up to 1 year postoperatively
5. Recommendations for follow-up treatment and rehabilitation
   Measuring time: During the hospital stay
6. Duration of rehabilitation / follow-up treatment duration
   Measurement duration: up to 1 year postoperatively
7. Diagnoses and organ complications
   Measurement duration: up to 1 year postoperatively
8. Concomitant medication
   Measurement duration: up to 1 year postoperatively
9. Utilization of health care services
   Measurement duration: up to 1 year postoperatively
10. Re-admissions
    Measurement duration: up to 1 year postoperatively
11. Additional costs of prehabilitation compared to standard care
    Measuring time: from randomisation to surgery

6.2.2 Final goals in the intervention group:

1. Satisfaction with the SDM conference (PEF-9)
2. Satisfaction with the intervention (ZUF 8)
3. Feedback questionnaire on nutritional councelling
4. Nutritional protocols

6.2.3 Influencing parameters

- Demographic data: age, gender, height, weight
- Type of operation
- Duration of anaesthesia
- Mini-Cog™ (paper-based cognition test) (indicates a pre-existing cognitive impairment)
- Serum albumin
- Haemoglobin and anaemia diagnostics

7 Study Structure

7.1 Study design

A multicentre, national, randomised, pragmatic, controlled, outcome assessor-blinded trial. The randomisation takes place as a two-arm parallel group design with an allocation ratio of 1:1 per hospital.

7.2 Study population

1,400 patients (700 each in the intervention and control group) undergoing elective surgery and meeting all inclusion and no exclusion criteria.

7.3 Inclusion and exclusion criteria

7.3.1 Inclusion criteria of the study group

1. Age ≥ 70 years
2. Patient able to provide consent or a legal representative for patients who are unable to provide consent
3. Planned elective surgery
4. Expected anaesthesia duration ≥60 min
5. Insured by a statutory health insurance *
6. Frailty syndrome (≥1 positive out of 5 standardised parameters according to the physical frailty phenotype (Fried et al., 2001))

* Legal basis selective contract with the insurance company, acc. §140a SGB V [Sozialgesetzbuch Fünftes Buch (Social Code Fifth Book)]; an alternative legal basis, following approval by the project management and project funding agency, is the §630 BGBa [Bürgerliches Gesetzbuch (Civil Code)], should no selective contracts exist.

7.3.2 Exclusion criteria of the study group

1. Severe cardiac or pulmonary disease (NYHA IV, Gold IV)
2. Intracranial interventions
3. Moribund patients (palliative situation)
4. Language barrier
5. No legally valid declaration of consent
6. Participation in another interventional rehabilitation study or an AMG or MPG study that was not approved by the project management. (Exception: parallel participation in adjuvant therapy study).

7.3.3 Descriptive controls (optional)

Inclusion and exclusion criteria analogous to the study participants, but with patients without frailty syndrome (no positive parameter of 5 standardised parameters according to the Physical Frailty Phenotype (Fried et al., 2001)).

7.3.4 Family member

Inclusion criteria: Regular (at least 1x / week) involvement in care, nursing or household management of the relative who was included in the study.

Exclusion criteria: insufficient language skills.

7.3.5 NCD control group (optional)

Inclusion criteria:

- Male and female patients aged 70-100 years, including controls from the POCD register (EA1 / 104/16).
- Healthy volunteers (ASA I) / ASA II + III who are not scheduled for surgery within the next year

Exclusion criteria:

- Surgery in the last six months before inclusion in this study
- Lack of consent
- Unwillingness to participate in the follow-up examinations or to be contacted for appointments
- Patients with a neuropsychiatric clinical presentation that limits the execution of neurocognitive tests.
- Patients with hearing and / or visual impairment or relevant language barriers that limit the execution of the neurocognitive tests
- Participation in another interventional rehabilitation study or an AMG or MPG study that was not approved by the project management.

7.4 Participation in other studies

Participation in another interventional rehabilitation study is not permitted. Participation in an AMG or MPG study is only permitted after examination and approval by the project management (exception: parallel participation in adjuvant therapy study is permitted).

7.5 Recruitment

The study participants are recruited in the participating hospitals.

After determining an elective surgery indication, all patients who meet the inclusion criteria 1-5 and no exclusion criteria are screened for the presence of a frailty syndrome (V0). If frailty or pre-frailty are identified, the patient is invited to participate in the study along with detailed information about the project. The patient confirms his / her participation in the selective contract by signing the declaration of participation and declaration of consent for the new healthcare form; by signing the consent to data processing for research purposes, the patient also agrees to the processing of research data for evaluation of the healthcare feature (V1).

7.6 Course of the study

After inclusion in the study, a baseline assessment takes place (V2, see “Organisation of visits“, p. 23). Following this examination, the patient is randomly assigned (1:1) to either the intervention group or the control group. An online randomisation tool is used for this purpose (see “Randomisation“ p. 24). Accordingly, the patients in the intervention group receive the planned interventions, consisting of participation in a joint interdisciplinary and interprofessional decision-making conference, as well as the subsequent three-week prehabilitation program.

Participants in the control group proceed with the planned operation, according to the standard of care, after completion of the baseline assessment. For the participants in the intervention group, the operation only takes place after the prehabilitation program. Both the operation and any subsequent rehabilitation programs take place according to the existing procedure catalogue. In addition, all participants are followed up postoperatively for 12 months (follow-up 1x / month).

The course of study is outlined in Fig. 1**Fehler! Verweisquelle konnte nicht gefunden werden.**.

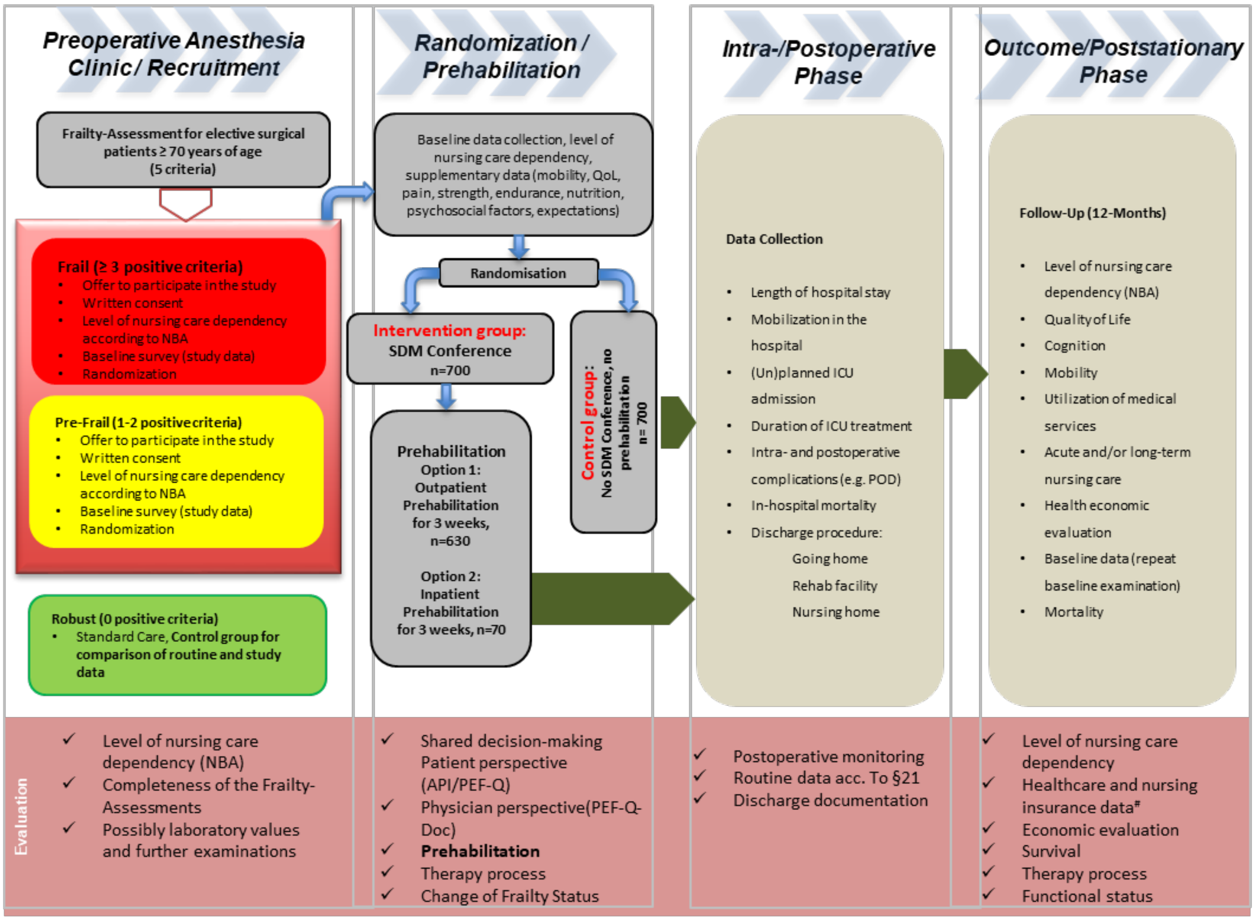

Fig. 1: Course of the study

7.7 Investigations and Assessments

7.7.1 Organisation of visits

The study is divided into 4 phases (screening and inclusion, shared decision making, prehabilitation and follow-up), during which a total of 18 visits take place (Table 1).

Table 1: Phases and visits in the planned evaluation of PRÄP-GO

Visit	Time	Description
**Phase I: Screening and inclusion**		
**V0**	Day 1	Frailty screening, mini-geriatric assessment
**V1**	Day 1	Study inclusion, master data collection
**V2**	Day 1	Basic assessment
**Phase II: Shared Decision Making**		
**V3**	Day 1-3	Organisation of SDM conference
**Phase III: Prehabilitation**		
**V4**	V3 + planning time[1]	Begin of prehabilitation
**V5**	V4 + 21 days	End of prehabilitation
**Phase IV: Follow-up**		
**V6**	3 - 14 days post-op	Hospital discharge
**V7, 8**	1st and 2nd month post-op	Short telephone interviews
**V9**	3rd month post-op	Detailed telephone interview / home visit
**V10 - V17**	4th – 11th month post-op	Short telephone interviews
**V18**	12 months post-op	Follow-up examination as a home visit

The contents of the examination times are described in Annex 2.

7.7.2 Classification of assessment procedures in the domains of the ICF

Based on the model of the International Classification of Functioning, Disability and Health (ICF), the assessment procedures used can be assigned to the different domains of health, social participation and

[1] Planning time depends on the availability of a prehabilitation place in the appropriate setting and the planned date of the operation. The goal is to plan the prehabilitation as soon as possible before the operation date.

independence in the activities of everyday life. In Table 2 can be seen that the selected assessment battery includes all domains of the ICF.

Table 2: Classification of assessment procedures in the domains of the ICF

<table>
<tr><th>Structure</th><th>Function</th><th>Activity</th><th>Social participation / participation</th><th>Environmental factors</th><th>Person-related factors</th></tr>
<tr><td>Level of damage
Laboratory</td><td>Mobility (TUG)
Grip strength
Walking speed
Malnutrition (MNA-SF)
Stair climbing ability
Cognition (Mini-Cog / MOCA MMQ / TMT A + B / CANTAB)
Anxiety (GAD-7)
Pain
Endurance (2-MST, peak flow)</td><td>2-MST
Falls</td><td>SOS
BSSS
PEF-FB-9</td><td></td><td>Weight
Height
Smoking (Fagerstrom)
Alcohol (AUDIT-C)
Co-Morbidities (CCI)
Autonomy requirements (API)
Fear of falling (ABC-6)</td></tr>
<tr><td></td><td colspan="3">MET
NBA</td><td></td><td></td></tr>
<tr><td></td><td colspan="2">Barthel Index
CHARMI</td><td></td><td></td><td></td></tr>
<tr><td></td><td></td><td colspan="2">Quality of Life (EQ-5D-5L)
Depression (PHQ-8)
Loneliness (UCLA-3-items)
Activities in daily life (Lawton & Brody)
Subj. Health (WHODAS 2.0)</td><td></td><td></td></tr>
<tr><td></td><td></td><td colspan="3">Accessible living space (Life Space Assessment)</td><td></td></tr>
</table>

List of Abbreviations:

2-MST = 2-minute step test, API = Autonomy Preference Index, BSSS = Berliner Social Support Scale, CCI = Charlson Comorbidity Index, CHARMI = Charité Mobility Index, EQ-5D-5L = EuroQol quality of life questionnaire, GAD = Generalized Anxiety Disorder Scale, MET = metabolic equivalent tasks, MMQ = Multifactorial Memory Questionnaire, NBA = new nursing care grading assessment , PEF-FB = Participative Decision Making Questionnaire, PHQ = Patient Health Questionnaire (Depression), TUG = Timed Up & Go, TMT = Trail Making Test, TSG = Stair Climb Speed, UCLA = University of California Los Angeles, WHODAS= World Health Organization Disability Assessment Schedule

7.7.3 Evidence of the assessment procedures used

Annex 3 includes the measurement dimensions and target criteria of all assessment procedures used in PRÄP-GO are summarised, along with a brief description of the measurement procedure and existing evidence based on academic/scientific publications.

7.8 Randomisation

Randomisation takes place after the baseline visit (V2, see “Organisation of visits“ p. 23). Allocation to prehabilitation and standard therapy is done in a 1:1 ratio, stratified by participating centres. The randomisation lists are created by an independent party at the Institute for Medical Information Processing, Biometry and Epidemiology at the Ludwig Maximilians University in Munich and kept

confidential throughout the study. A web-based tool is used for randomisation Research Electronic Data Capture Software (REDCap). An independent party at each participating hospital carries out the randomisation via online access and informs the patient, the physician in charge and the study management of the randomisation result.

Randomisation is performed 1:1 into 2 groups:

1) An intervention group, which will receive a decision-making conference and take part in a prehabilitation program.
2) A control group, which will receive standard of care, without a decision-making conference or prehabilitation program.

7.9 Intervention

7.9.1 General Description

The intervention in the PRÄP-GO project consists of two parts. In the first part, a shared decision making conference is carried out in order to decide, together with the patient and all involved disciplines, about the type and goals of the pre-rehabilitation intervention. In the second part, the prehabilitation intervention is carried out. These two parts of the intervention are detailed below.

7.9.2 Shared Decision Making (SDM) conference

In the PRÄP-GO project, the so-called "Three Talks Model" (TTM) is used to conduct the SDM conference (Elwyn et al. 2017). However, due to the special circumstances during the course of the study, the implementation must be adapted (Fig. 2).

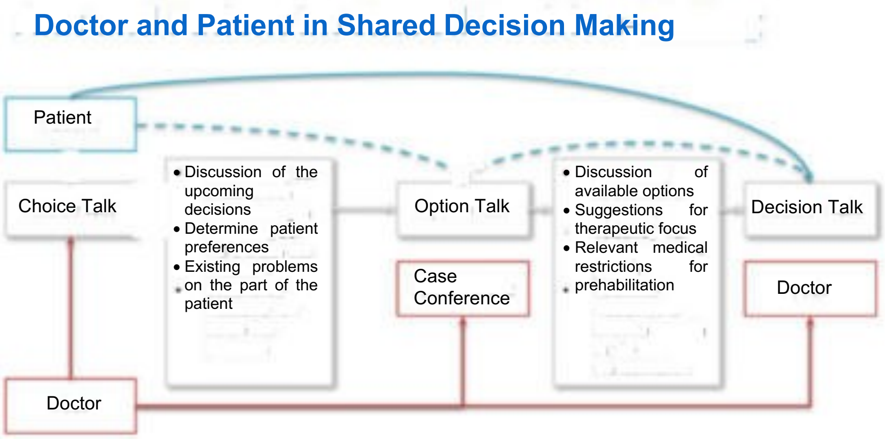

Fig. 2: Application of the three-talk model in the PRÄP-GO project
(Author's depiction)

The first and third phases of the SDM process take place in a conversation between the patient and a physician.

In the first phase (choice talk), the patient is prepared for the joint decision-making process and preferences regarding the patient's extent of participation in the decision-making process are determined. In addition, patient questions and issues are gathered for discussion in the subsequent case conference, and any initial preferences regarding the conduction of the prehabilitation are determined. This should also include psychosocial factors that influence the setting and goals within prehabilitation. Depending on the patient's participation preferences, physician and patient can also agree on subject areas for which the physician should act as proxy for the patient's interests during the case conference.

The second phase (Option Talk) takes place as a case conference, in which, in addition to the initial physician who performed the choice talk, other representatives of medical specialties (anaesthesiology, surgical specialty, geriatrics) as well as a therapist (physiotherapist, occupational therapist) and a nurse should be present. The aim of this SDM conference is to determine the design (outpatient / inpatient / semi-inpatient) as well as the primary and secondary goals of the treatment. Therapy planning and implementation are then derived from the defined therapy goals.

The following professional representatives are intended to participate in the case conference:

1. Doctor of anaesthesiology (specialist standard; mandatory participation)
2. Surgeon (specialist standard; mandatory participation)
3. Specialist with additional training for geriatrics (or currently in training with a person authorised for continuing education according to § 5 para. 1 clause 2 of the Federal Medical Association for Geriatrics; mandatory participation)
4. Nurse or representative of the therapeutic professions (physiotherapist or occupational therapist) (a representative of these groups is mandatory)
5. General practitioner / family doctor (optional)

In addition to their own expertise, the doctor who accompanied the first phase also ensures that the patient's preferences and other factors identified during the first phase are taken into account. This is particularly relevant for patients that choose to have a limited or no participation in the conference.

The final part of the SDM process (Decision Talk) serves to summarise the results of the case conference and to make the upcoming decisions together with the patient. Both sides once again have the opportunity to ask and clarify final questions.

A family member may (optionally) take part in the patient's SDM procedure.

The details of the process and quality assurance are described in the SDM manual (Annex 4).

7.9.3 Prehabilitation

Definition of prehabilitation goals

In the SDM conference, one primary and two secondary therapy goals are formulated on the basis of the risk factors identified in the previous assessments in order to reduce the impact of these risks. These

areas are to be strengthened by improving bio-psycho-social resources in order to avoid perioperative complications and deterioration of nursing care dependency level.

The formulated therapy goals serve as the basis for the therapeutic intervention planning of the prehabilitation program. For this reason, it is important to formulate the therapy goals in such a way that they relate to the various forms of intervention in prehabilitation. Conversely, it is equally important to formulate the goals of prehabilitation in such a way that their relevance is clear to the patient. Goals formulated by a patient are seldom based on functional requirements, as shown by clinical assessments, but generally on activities and / or aspects of social participation that are relevant to them. For this reason, the objectives should be specified further and reflect the everyday goals of the patient. An objective, such as "improving strength", is not sufficient and may not be understood by the patient. Instead, the functional objective ("improvement of strength") should be combined with a defined everyday activity that requires improvement or that is to be learned (anew). This everyday activity should always be agreed with the patient as part of SDM decision making, so as to ensure that the overall objective is understandable and relevant for the patient.

Additional goals may be set based on the risk areas identified during the baseline assessment and that should be reduced or eliminated as part of the prehabilitation. These clinical goals can either take place independently (for example, a separate diagnosis) of the (training therapy) prehabilitative intervention or can go hand in hand with them (for example, a functional deficit that is not targeted by the planned operation).

Examples of different therapy goals and their specifications are shown in Table 3.

Table 3: Intervention forms and therapy goals

Form of assistance	Therapy goal	Examples of specifications
**Strength training**	Strength improvement	Improvement of standing and walking ability.<br>Improve trunk stability as preparation for walking on crutches.
**Endurance training**	Improve aerobic endurance	Ability to walk continuously for at least 30 minutes.
**Balance / mobilisation training**	Improving balance<br>Improving mobility	Reduction of the risk of falling<br>Improving the ability to climb stairs<br>Improvement of joint mobility (possibly specifying which joints)
**Everyday training**	Improving everyday skills	Training in the preparation of meals<br>Shopping training

Form of assistance	Therapy goal	Examples of specifications
**Nutritional counselling***	Improving the nutritional situation	Eliminating malnutrition<br>Elimination of Ca^{2+}-deficiency<br>Weight loss of 5 kg
**Speech therapy**	Improvement of speech/speaking ability or difficulties swallowing	Improved swallowing ability to help eliminate malnutrition
**Respiratory therapy***	Improvement of O_2-intake	Learning to work with a breathing trainer
**Support training***	Improvement of mobility under partial weight-bearing	Learning to walk on crutches under partial weight-bearing
**Massages***	Relieving painful muscle tension	Specification of the affected muscle groups
**Social counselling***	Identification of social problems<br>Taking appropriate measures to alleviate / avoid social problems	Referral to a family doctor<br>Request an assessment by the MdK [Medizinischer Dienst der Krankenversicherung (Medical service of the health insurance)] in order to ascertain a nursing level of care
**Cognitive training***	Learning cognitive strategies	Training of memory strategies<br>Biography work
**Medical objectives**	Reduction of polypharmacy	Review and adjustment of the medication plan<br>Avoidance of polypharmacy

* These are therapies that take place at a lower therapy frequency than the other therapies and are therefore subsumed in the schedule under "Other" (see Table 4).

The SDM conference not only formulates the therapy goals, but also prioritises them. In this way, one primary and two secondary therapy goals are defined. The definition takes place within the framework of the SDM conference on the basis of the inclusion of patient goals, the degree of concern and the accessibility of realistic goals, the implementation of which can lead to a risk reduction. Here, too, the prioritisation of therapy goals should be agreed upon with the patient. This prioritisation has a significant impact on the therapy frequency (see below).

7.9.4 Training plan

The goal of training planning is to provide each patient with a training plan that is:

1. goal-directed
2. individually tailored
3. based on the rehabilitation goals formulated in the shared decision making (SDM) conference and
4. conceived and controlled based on evidence-based knowledge

.

Each patient receives 30 units of supervised therapy by a specialist within the 3 weeks of prehabilitation. Each session lasts 30 minutes; this corresponds to 2 therapy units (TU) every business day. The TUs carried out in one day can either take place together or separately.

In addition, each patient is asked to independently perform at least one additional TU every business day. An additional, independently performed TU per week is to be carried out for breathing training in preparation for the postoperative phase. For patients undergoing operation on the lower extremities, the use of crutches should also be trained as preparation for the postoperative phase. The use of these aids (crutches, breathing trainers) is practiced as part of the supervised therapy and should then be practiced independently by the patient.

In this way, each patient receives 48 units of targeted therapy according to the goals formulated in the SDM conference.

7.9.5 Schedule the training

The number of TUs that should be carried out within the 3 weeks of prehabilitation to achieve the primary and the two secondary rehabilitation goals is shown in Table 4. Planning is done individually for each week. This means that the training intensity is adjusted according to the previously achieved performance. Such adjustment can also be carried out individually by the responsible therapist in order to avoid overloading or underloading the patient and to ensure an optimal training stimulus.

Table 4: Time planning and training focus divided by week in prehabilitation

**Training plan**						
**Training Focus**	**Week 1**		**Week 2**		**Week 3**	
	**Sup**	**ST**	**Sup**	**ST**	**Sup**	**ST**
**Primary**	5	2	5	2	5	2
**Secondary 1**	3	2	2	2	2	2
**Secondary 2**	1	1	1	1	1	1
**Other**	1	1	2	1	2	1
**Total**	10	6	10	6	10	6

Abbr.: Sup: supervised training; ST: self training

7.9.6 Details of prehabilitation services and therapy

The details of the various interventions can be seen in the Annex 5 (training manual) and Annex 6 (nutrition counselling manual).

7.10 Follow-up

7.10.1 Description

Phase IV of the evaluation, shown in Table 1 (Table 1, p. 23), is defined as the follow-up phase. This phase begins with discharge from the hospital after the planned operation (V6) and ends 12 months after the day of surgery (V18). For organisational reasons, a time window of ± 14 days is provided for the final Visit 18. In addition, monthly follow-up examinations take place per telephone or as telemedical visits (V7, V8, V10-V17), which serve to maintain contact with the study participants and allow a short survey to take place. A more detailed survey is planned 3 months after discharge from the hospital (V9) as a home visit. If a home visit is not possible at this time, this visit may also be carried out by telephone / telemedicine. In the case of a home visit, the entire test battery of the baseline visit should be repeated; in the case of a telephone or telemedical visit, a shortened test battery will take place (see Table 5, p. 31).

The follow-up phase is used to analyse the short and long-term effects of prehabilitation and to answer related research questions, in particular the effects of prehabilitation in terms of preventing nursing care dependency after elective surgery.

7.10.2 Visit 6

As part of the hospital discharge, the following data is transmitted from the operating hospital to the study centre:

1. Data delivery according to §21 KHEntgG [Krankenhausentgeltgesetz (Hospital Remuneration Act)]
2. Intensive care unit reports
3. Discharge report
4. Discharge medication (standardized federal medication plan)

7.10.3 Visits 7, 8, 10 - 17

Visits 7, 8 and 10 to 16 consist of short telephone interviews. These take place once a month. The interviews will cover any falls that occurred in the previous month, the current medication, current pain and the use of medical, nursing or therapeutic services. In addition, Visit 7 will include questions regarding the type of follow-up treatment required after discharge from the hospital (rehabilitation, follow-up treatment, outpatient treatment) and its duration. The exact contents of the follow-up visits are shown in Table 5.

Table 5: Data collected during telephone follow-up surveys

Visit	V7, V8	V9	V10-17
**Type**	Telemedical	Home visit / telemedical	Telemedical
**Time**	1. + 2. month post-op	3rd month post-op	4. – 11. month post-op
**Assessments and examinations**	Duration of rehabilitation / further therapy (only V7)<br>Number of falls<br>Number / type of medication<br>Pain<br>Activities of daily living<br>Current supportive equipment	**Home visit:**<br>Baseline visit battery (V2)<br>Use of medical services<br><br>**Telemedical:**<br>As V8<br>current level of nursing care dependency and assistance<br>NBA<br>Use of medical services<br>Living situation<br>Life Space Ass.<br>ABC-6<br>Quality of life<br>WHODAS 2.0<br>PHQ-8<br>GAD-7	Number of falls<br>Number / type of medication<br>Pain<br>Activities of daily living<br>Living situation (V12, V15<br>Current supportive equipment<br>Use of medical services (only V12, V15)<br>Quality of life (V12, V15)<br>WHODAS 2.0 (V12, V15)

7.10.4 Visit 9

Visit 9 takes place three months after the operation and should be carried out as a home visit. If not possible, a telemedical or telephone visit may take place.

If the appointment takes place as a home visit, in addition to all the information requested in visit 8, a re-evaluation of level of nursing care dependency is carried out using the NBA, and the actual existing level of nursing care will be documented. Furthermore, the V2 assessment of LSA, ABC scale, EQ5D-5L, PHQ 8 , GAD-7 and WHODAS 2.0 will be performed once again. In case of a conspicuous MiniCog resulting in an extended cognitive test battery (MiniCog, MOCA, Animal Naming Test, Trail Making Test A and B, NCD Diagnostics) including CANTAB at V2, this test battery will again be carried out during this home visit.

A telemedical or telephone visit is carried out akin to the Visit 8.

7.10.5 Visits 12 and 15

A new health economic analysis is carried out for visits 12 and 15. For this purpose, the FIMA questionnaire is queried again (with the exception of the constant characteristics of the patient), along with the EQ-5D-5L and WHODAS 2.0.

7.10.6 Visit 18

Visit 18 takes place as a home visit for all included study participants. During this visit, a final assessment of the nursing care dependency level is carried out by means of the NBA. In addition, all assessments initially recorded in visits 0 and 2 are repeated, so as to identify trends within the two study groups, as well as comparative differences.

8 Ethics

There is an ethical vote by the Charité - Universitätsmedizin Berlin.

The cooperation clinics obtain a corresponding ethics vote from the responsible ethics committee before the start of the study participation. A copy of the vote must be sent to the Charité study centre.

Amendments to the current version of the ethics vote will be communicated to all cooperation partners by the study centre during the planning phase. After issuing a new vote, the cooperation partners submit the amendment to the ethics committee responsible for them as soon as possible.

If a cooperation clinic submits a supplementary amendment that is only valid for them to the appropriate ethics committee, this must also be communicated to the Charité study centre during the planning phase. A copy of the ethics vote of the supplementary amendment must also be sent to the study centre. Further cooperation clinics have the opportunity to join this supplement by submitting their own amendments.

9 Data management

9.1 Collection methods

All data is collected by trained personnel at each participating location, and documented using a Web eCRF database. The research staff enter the data directly on site (exception: in the prehabilitation clinics, data can be collected on paper CRFs. Paper CRFs are always available as fall-back documentation for the eCRFs; however, the data collected in paper CRFs must be later entered in the eCRF database by the study staff on site). Data queries can be generated automatically.

Study day 1 begins with the written consent and ends with the end of the calendar day at 11:59 p.m. Patients who have consented will be followed up to death or until the last visit (day 365 ± 14 days after elective surgery), whichever comes first. Complete log data is collected for all consenting patients, including those who are eventually excluded. If consent to participate has been withdrawn, the data will not be used unless consent has been provided.

9.2 Data management

Data management is coordinated by the project management at the Charité, including programming (online study database design) and support in data management (including data monitoring, database questions, technical questions, data queries, query resolution).

9.3 Data quality and monitoring

Several methods were employed to ensure data quality, and standardisation via protocol helps prevent distortion and optimise data quality. Mechanisms used are:

- Start-up meeting for all participating centres, which will be held before the start of the study to ensure the consistency of the procedures
- A detailed data dictionary will define the data to be recorded and entered in the eCRF
- Automated control of data entry in the eCRF
- The coordination centre will carry out regular validation of the data, requesting clarifications or corrections as needed

The study is monitored by quality control checks of protocol compliance and data queries. The coordination centre may carry out on-site monitoring for this purpose.

Quality control issues will be identified through the electronic data checks or during a site audit visits.

9.4 Protocol deviations

A deviation of protocol is an unforeseen or unintentional change from the expected conduction of an approved study that does not match the current study protocol or the consented process. A deviation of protocol can be an omission, an error, an addition, or a change in a procedure described in the protocol.

As the examiner is responsible for patient safety and diligence in performing the exam, he / she may deviate from the protocol to avert an immediate threat to study patients.

- All protocol deviations are to be reported immediately using eCRF, analog fax or digital scan.
- A report on protocol deviations should be available in the study database.

10 Statistics

10.1 Power calculation and sample size

Based on available literature (Table 7 on page 789 (Müller-Mai et al. 2015)), the following assumptions can be made (see Table 6) regarding changes in the level of nursing care dependency expected in the intervention group (prehabilitation) and the control group (current clinical standard):

Table 6: Quantification of the intervention effect based on Müller-Mai et al. (2015)

Change in care levels	Intervention (prehabilitation)	Control (current clinical standard)
**Improvement by ≥ 1 level**	5%	2.5%
**Patient remains on the same level**	47.5%	42.5%
**1 level deterioration**	45%	50%
**Deterioration by ≥ 2 levels**	2.5%	5%
**Total**	100%	100%

The occurrence probabilities of the categories mentioned formally considered ordinally scaled outcomes. Based on this, the number of cases was calculated using nQueryAdvisor V7 (MTT2-tmpB3E4 modules) (Kolassa 1995).

Given the quantified effects between intervention and control groups shown in Table 6, a difference between the two groups can be demonstrated at a bilateral 5% significance level and with a power of 80% if 470 patients are analysed per group. Assuming 2.5% incorrect treatment allocation and 30% loss to follow-up up to Visit 18, the total number of cases to be included is 1378 participants. With a total of 13 recruiting centres (Charité: n = 200; 12 further cooperation partners á n = 100), there would be a total of n = 1400 participants. If one cooperation partner were to fail completely (-100 study participants), the remaining 12 centres (plus n = 7 per centre) could compensate for the failure to return to a total of 1384 study participants (207 + 11 × 107).

The inclusion of 1,400 patients is therefore planned.

10.2 Analysis of the results

The joint federal committee stipulates that the evaluation must be carried out independently as a primary task of one of the consortium partners. Accordingly, the statistical evaluation will be carried out by the Institute for Medical Information Processing, Biometry and Epidemiology, Medical Faculty of the Ludwig Maximilians University, Munich. The health economic analyses will be carried out by the TU

Berlin. Further explorative and secondary analyses, as well as accompanying research, can also be carried out by other cooperation partners, such as the Institute for Biometry and Clinical Epidemiology at the Charité.

All statistical analyses are carried out after the data collection phase is completed. A detailed statistical analysis plan (SAP) will be prepared before the study database is completed.

Descriptive data analyses take place after completion of the baseline survey. They are carried out for the entire sample as well as stratified by frailty status. This is intended to provide a first impression of the distribution within the strata with regard to the relevant outcomes.

The mean and standard deviation are shown for continuous variables, and relative and absolute frequencies for categorical variables. Plausibility checks are carried out before the data analysis. The level of significance is set at 0.05. The p-value is intended to give the measure of the strength of the association between the dependent and the independent variables.

10.2.1 Primary endpoint

The aim of the study is to confirm the effectiveness of prehabilitation program. This will be done by evaluating the change in level of nursing care dependency according to the new nursing care grading assessment (NBA) (Wingenfeld, Büscher, and Gansweid 2008), which is defined as the primary end point.

The evaluation of the primary end point - change in the level of nursing care dependency according to the new nursing care grading assessment – will be carried out using the Wilcoxon-Mann-Whitney rank sum test. This test also allows easy stratification. Models for multivariate ordinal regression are used as sensitivity analysis (Hirk, Hornik, and Vana 2017). These allow adjustment for relevant confounders at the time of the basic survey, as well as the consideration of longitudinal data and their correlation structures. If a patient dies, the fifth level of care will be assigned, which indicates the highest level of care dependency.

10.2.2 Secondary endpoints

Secondary endpoints - frailty status, cognition, health-related quality of life, survival, etc. - are compared between the groups at the time of the survey.

Incidences of postoperative complications between the groups are analysed descriptively and compared with current literature (Birkelbach et al. 2017).

Different health economic parameters are compared between the groups. The main analysis of the health economics evaluation is a cost-utility analysis (CUA) from a social perspective. The costs include the additional costs of prehabilitation compared to standard care and the direct and indirect medical costs incurred in the groups over the study period. The benefit is primarily depicted with the help of quality-adjusted years of life (QALYs), which are determined from the survival and the benefit-weighted quality

of life of the patients (EQ-5D-5L). First, an incremental cost-effectiveness ratio (IKER, i.e. additional costs per additional QALY) is calculated and then a cost-effectiveness acceptance curve is created to check the probability with which the IKER can be considered cost-effective under various assumptions about willingness to pay. In addition, the costs are also related to other endpoints (including nursing care dependency level, survival, WHO DAS). The uncertainty in the results is examined using probabilistic and deterministic sensitivity analyses.

The Shared Decision Making Conference (SDM) will also be evaluated. Decision-making satisfaction is examined from a patient and physician perspective both pre- and post-SDM, as well as the Autonomy Preference Index (API) (Simon et al. 2010). An exploratory analysis will investigate whether participation in decision-making conferences leads to a greater satisfaction the decisions and reduce decision-making conflicts with the patient (PEF-FB 9).

11 Safety

11.1 Adverse events

Adverse events (AEs) are defined as any adverse medical event in a patient or clinical subject-matter that has undergone an examination intervention. Such an event does not necessarily have a causal relation to the intervention. The underlying mortality and morbidity rate of patients participating in the trial is high. Common aberrations in laboratory values, signs and symptoms due to the underlying illness and the impact of standard therapies will not necessarily constitute an adverse event unless they are considered to be of concern or related to the study or the intervention in the investigator's clinical judgement.In this case, an adverse event (AE) should be documented.

Adverse events will be monitored and documented from the time of consent up to 48 hours after the end of the study. The patient should be observed until the event is resolved or explained (day 365 ± 14 days after the elective surgery). The frequency of the follow-up assessments in this regard is at the discretion of the person responsible for the respective study centre.

11.2 Safety

The mortality rate of the patients participating in the studies is high. Therefore, events that are or are expected to be part of the natural course of the primary disease process are not reported as serious adverse events in this study.

In particular, events that have already been defined and reported as study results (e.g. mortality, intensive care) will not be reported separately as adverse or serious adverse events unless they are considered to be causally related to the study intervention.

The following events are definitely reported as SAE:

- Fall to the ground during the basic assessment or a home visit (each time from the start of the assessment until 48 hours afterwards)
- During the prehabilitation phase (start of the first therapy unit until 48 hours after the last unit)
    - Unplanned inpatient hospital admission
    - Unplanned intervention (e.g. cardiac catheter)
    - Unplanned surgery
    - Cardiac arrest
    - Fall to the ground

11.3 Reporting

Adverse events are recorded in the eCRF. All serious adverse events must be reported to the coordination centre through the database within 72 hours after the auditor notifies them of the event.

Contact telephone numbers for SAE advice = +49 (0)30 - 450 631245

12 Other

12.1 Financing of the study

The new healthcare feature is funded by the Innovation Committee of the Federal Joint Committee based in Berlin (grant number 01NVF18024; also see: https://innovationsfonds.g-ba.de/projekte/neue-versorgungsformen/praep-go-praehabilitation-von-aelteren-patients-with-frailty-before-elective-operations.276).

12.2 Publication guidelines

All publications resulting from the planning, implementation and analysis of the study described here are carried out according to academic and scientific standards, and will be submitted only in academic/scientific journals with a proven peer review process.

The standards of the Equator Network apply to all publications (https://www.equator-network.org/).

12.3 Registration of the study

The study will be registered on ClinicalTrials.gov before patient recruitment begins. The descriptions of prospective secondary analyses should be published before the database closes (e.g. in stat. analysis plan).

13 Literature/References

Birkelbach, Oliver, Rudolf Mörgeli, Felix Balzer, Maria Olbert, Sascha Treskatsch, Rainer Kiefmann, Ursula Müller-Werdan, et al. 2017. "[Why and How Should I Assess Frailty? A Guide for the Preoperative Anesthesia Clinic]." *Anasthesiologie, Intensivmedizin, Notfallmedizin, Schmerztherapie: AINS* 52 (11–12): 765–76. https://doi.org/10.1055/s-0043-104682.

Birkelbach, Oliver, Rudolf Mörgeli, Claudia Spies, Maria Olbert, Björn Weiss, Maximilian Brauner, Bruno Neuner, Roland C. E. Francis, Sascha Treskatsch, and Felix Balzer. 2019. "Routine Frailty Assessment Predicts Postoperative Complications in Elderly Patients across Surgical Disciplines - a Retrospective Observational Study." *BMC Anesthesiology* 19 (1): 204. https://doi.org/10.1186/s12871-019-0880-x.

Chen, Huifen, Suyun Li, Tingyu Ruan, Li Liu, and Li Fang. 2018. "Is It Necessary to Perform Prehabilitation Exercise for Patients Undergoing Total Knee Arthroplasty: Meta-Analysis of Randomized Controlled Trials." *The Physician and Sportsmedicine* 46 (1): 36–43. https://doi.org/10.1080/00913847.2018.1403274.

Deschodt, Mieke, Tom Braes, Johan Flamaing, Elke Detroyer, Paul Broos, Patrick Haentjens, Steven Boonen, and Koen Milisen. 2012. "Preventing Delirium in Older Adults with Recent Hip Fracture through Multidisciplinary Geriatric Consultation." *Journal of the American Geriatrics Society* 60 (4): 733–39. https://doi.org/10.1111/j.1532-5415.2012.03899.x.

Elwyn, Glyn, Marie Anne Durand, Julia Song, Johanna Aarts, Paul J. Barr, Zackary Berger, Nan Cochran, et al. 2017. "A Three-Talk Model for Shared Decision Making: Multistage Consultation Process." *BMJ (Clinical Research Ed.)* 359 (November): j4891. https://doi.org/10.1136/bmj.j4891.

Fried, L. P., C. M. Tangen, J. Walston, A. B. Newman, C. Hirsch, J. Gottdiener, T. Seeman, et al. 2001. "Frailty in Older Adults: Evidence for a Phenotype." *The Journals of Gerontology. Series A, Biological Sciences and Medical Sciences* 56 (3): M146-156.

Friedman, Susan M., Daniel A. Mendelson, Karilee W. Bingham, and Stephen L. Kates. 2009. "Impact of a Comanaged Geriatric Fracture Center on Short-Term Hip Fracture Outcomes." *Archives of Internal Medicine* 169 (18): 1712–17. https://doi.org/10.1001/archinternmed.2009.321.

Gill, Thomas M., Dorothy I. Baker, Margaret Gottschalk, Peter N. Peduzzi, Heather Allore, and Peter H. Van Ness. 2004. "A Prehabilitation Program for the Prevention of Functional Decline: Effect on Higher-Level Physical Function." *Archives of Physical Medicine and Rehabilitation* 85 (7): 1043–49. https://doi.org/10.1016/j.apmr.2003.10.021.

Gillis, Chelsia, Katherine Buhler, Lauren Bresee, Francesco Carli, Leah Gramlich, Nicole Culos-Reed, Tolulope T. Sajobi, and Tanis R. Fenton. 2018. "Effects of Nutritional Prehabilitation, With and Without Exercise, on Outcomes of Patients Who Undergo Colorectal Surgery: A Systematic Review and Meta-Analysis." *Gastroenterology* 155 (2): 391-410.e4. https://doi.org/10.1053/j.gastro.2018.05.012.

Gurlit, Simone, and Manfred Gogol. 2019. "Prehabilitation Is Better than Cure." *Current Opinion in Anaesthesiology* 32 (1): 108–15. https://doi.org/10.1097/ACO.0000000000000678.

Gustafson, Y., B. Brännström, D. Berggren, J. I. Ragnarsson, J. Sigaard, G. Bucht, S. Reiz, A. Norberg, and B. Winblad. 1991. "A Geriatric-Anesthesiologic Program to Reduce Acute Confusional States in Elderly Patients Treated for Femoral Neck Fractures." *Journal of the American Geriatrics Society* 39 (7): 655–62.

Hirk, Rainer, Kurt Hornik, and Laura Vana. 2017. "Mvord: An R Package for Fitting Multivariate Ordinal Regression Models." https://CRAN.Rproject.org/package=mvord.

Jahic, Dzenan, Djemil Omerovic, Adnana Talic Tanovic, Fuad Dzankovic, and Merita Tiric Campara. 2018. "The Effect of Prehabilitation on Postoperative Outcome in Patients Following Primary Total Knee Arthroplasty." *Medical Archives (Sarajevo, Bosnia and Herzegovina)* 72 (6): 439–43. https://doi.org/10.5455/medarh.2018.72.439-443.

Kim, Sun-wook, Ho-Seong Han, Hee-won Jung, Kwang-il Kim, Dae Wook Hwang, Sung-Bum Kang, and Cheol-Ho Kim. 2014. "Multidimensional Frailty Score for the Prediction of Postoperative Mortality Risk." *JAMA Surgery* 149 (7): 633–40. https://doi.org/10.1001/jamasurg.2014.241.

Kolassa, J. E. 1995. "A Comparison of Size and Power Calculations for the Wilcoxon Statistic for Ordered Categorical Data." *Statistics in Medicine* 14 (14): 1577–81.

Labra, Carmen de, Christyanne Guimaraes-Pinheiro, Ana Maseda, Trinidad Lorenzo, and José C. Millán-Calenti. 2015. “Effects of Physical Exercise Interventions in Frail Older Adults: A Systematic Review of Randomized Controlled Trials.” *BMC Geriatrics* 15 (December): 154. https://doi.org/10.1186/s12877-015-0155-4.

Leung, Jacqueline M., Tiffany L. Tsai, and Laura P. Sands. 2011. “Brief Report: Preoperative Frailty in Older Surgical Patients Is Associated with Early Postoperative Delirium.” *Anesthesia and Analgesia* 112 (5): 1199–1201. https://doi.org/10.1213/ANE.0b013e31820c7c06.

Lin, Hui-Shan, J. N. Watts, N. M. Peel, and R. E. Hubbard. 2016. “Frailty and Post-Operative Outcomes in Older Surgical Patients: A Systematic Review.” *BMC Geriatrics* 16 (1): 157. https://doi.org/10.1186/s12877-016-0329-8.

Lorenzo-López, Laura, Ana Maseda, Carmen de Labra, Laura Regueiro-Folgueira, José L. Rodríguez-Villamil, and José C. Millán-Calenti. 2017. “Nutritional Determinants of Frailty in Older Adults: A Systematic Review.” *BMC Geriatrics* 17 (1): 108. https://doi.org/10.1186/s12877-017-0496-2.

Ludwig, Kristina, J.-Matthias Graf von der Schulenburg, and Wolfgang Greiner. 2018. “German Value Set for the EQ-5D-5L.” *PharmacoEconomics* 36 (6): 663–74. https://doi.org/10.1007/s40273-018-0615-8.

Martin, Emily T., Keith S. Kaye, Caitlin Knott, Huong Nguyen, Maressa Santarossa, Richard Evans, Elizabeth Bertran, and Linda Jaber. 2016. “Diabetes and Risk of Surgical Site Infection: A Systematic Review and Meta-Analysis.” *Infection Control and Hospital Epidemiology* 37 (1): 88–99. https://doi.org/10.1017/ice.2015.249.

Moyce, Z., R. N. Rodseth, and B. M. Biccard. 2014a. “The Efficacy of Peri-Operative Interventions to Decrease Postoperative Delirium in Non-Cardiac Surgery: A Systematic Review and Meta-Analysis.” *Anaesthesia* 69 (3): 259–69. https://doi.org/10.1111/anae.12539.

———. 2014b. “The Efficacy of Peri-Operative Interventions to Decrease Postoperative Delirium in Non-Cardiac Surgery: A Systematic Review and Meta-Analysis.” *Anaesthesia* 69 (3): 259–69. https://doi.org/10.1111/anae.12539.

Müller-Mai, C. M., U. S. Schulze Raestrup, T. Kostuj, G. Dahlhoff, C. Günster, and R. Smektala. 2015. “[One-year outcomes for proximal femoral fractures: Posthospital analysis of mortality and care levels based on health insurance data].” *Der Unfallchirurg* 118 (9): 780–94. https://doi.org/10.1007/s00113-013-2534-7.

Rana, Maunak V., Lara K. Bonasera, and Gregory J. Bordelon. 2017. “Pharmacologic Considerations of Anesthetic Agents in Geriatric Patients.” *Anesthesiology Clinics* 35 (2): 259–71. https://doi.org/10.1016/j.anclin.2017.01.011.

Saczynski, Jane S., Edward R. Marcantonio, Lien Quach, Tamara G. Fong, Alden Gross, Sharon K. Inouye, and Richard N. Jones. 2012. “Cognitive Trajectories after Postoperative Delirium.” *The New England Journal of Medicine* 367 (1): 30–39. https://doi.org/10.1056/NEJMoa1112923.

Santos-Eggimann, Brigitte, Patrick Cuénoud, Jacques Spagnoli, and Julien Junod. 2009. “Prevalence of Frailty in Middle-Aged and Older Community-Dwelling Europeans Living in 10 Countries.” *The Journals of Gerontology. Series A, Biological Sciences and Medical Sciences* 64 (6): 675–81. https://doi.org/10.1093/gerona/glp012.

Sepehri, Aresh, Thomas Beggs, Ansar Hassan, Claudio Rigatto, Christine Shaw-Daigle, Navdeep Tangri, and Rakesh C. Arora. 2014. “The Impact of Frailty on Outcomes after Cardiac Surgery: A Systematic Review.” *The Journal of Thoracic and Cardiovascular Surgery* 148 (6): 3110–17. https://doi.org/10.1016/j.jtcvs.2014.07.087.

Sibia, Udai S., Adam S. Weltz, James H. MacDonald, and Paul J. King. 2018. “Insulin-Dependent Diabetes Is an Independent Risk Factor for Complications and Readmissions After Total Joint Replacements.” *Journal of Surgical Orthopaedic Advances* 27 (4): 294–98.

Simon, Daniela, Levente Kriston, Andreas Loh, Claudia Spies, Fueloep Scheibler, Celia Wills, and Martin Härter. 2010. “Confirmatory Factor Analysis and Recommendations for Improvement of the Autonomy-Preference-Index (API).” *Health Expectations: An International Journal of Public Participation in Health Care and Health Policy* 13 (3): 234–43. https://doi.org/10.1111/j.1369-7625.2009.00584.x.

Smilowitz, Nathaniel R., Navdeep Gupta, Yu Guo, Joshua A. Beckman, Sripal Bangalore, and Jeffrey S. Berger. 2018. “Trends in Cardiovascular Risk Factor and Disease Prevalence in Patients

Undergoing Non-Cardiac Surgery.” *Heart (British Cardiac Society)* 104 (14): 1180–86. https://doi.org/10.1136/heartjnl-2017-312391.

Swank, Ann M., Joseph B. Kachelman, Wendy Bibeau, Peter M. Quesada, John Nyland, Arthur Malkani, and Robert V. Topp. 2011. “Prehabilitation before Total Knee Arthroplasty Increases Strength and Function in Older Adults with Severe Osteoarthritis.” *Journal of Strength and Conditioning Research* 25 (2): 318–25. https://doi.org/10.1519/JSC.0b013e318202e431.

Watt, Jennifer, Andrea C. Tricco, Catherine Talbot-Hamon, Ba’ Pham, Patricia Rios, Agnes Grudniewicz, Camilla Wong, Douglas Sinclair, and Sharon E. Straus. 2018. “Identifying Older Adults at Risk of Harm Following Elective Surgery: A Systematic Review and Meta-Analysis.” *BMC Medicine* 16 (1): 2. https://doi.org/10.1186/s12916-017-0986-2.

Wingenfeld, K, A Büscher, and B Gansweid. 2008. “Das neue Begutachtungsassessment zur Feststellung von Pflegebedürftigkeit.” Bielefeld / Münster: Institut für Pflegewissenschaft an der Universität Bielefeld (IPW) / Medizinischer Dienst der Krankenversicherung Westfalen-Lippe (MDK WL). https://www.uni-bielefeld.de/gesundhw/ag6/downloads/Abschlussbericht_IPW_MDKWL_25.03.08.pdf.

Annexes

Annex 1: DSMC Charter

Annex 2: Table of the assessment procedures to be carried out for the various visits

Annex 3: Measuring instruments used, including evidence

Annex 4: SDM manual

Annex 5: Training manual

Annex 6: Nutritional manual
