# Supplementary material for: Prehabilitation of elderly frail or pre-frail patients prior to elective surgery (PRAEP-GO): study protocol for a randomized, controlled, outcome assessor-blinded trial
Source: Trials. 2022 Jun 6;23:468. doi: 10.1186/s13063-022-06401-x (PMC9167908; doi:10.1186/s13063-022-06401-x)
Supplement: Supplementary file 7 — Additional file 7. Ethical vote. [file 13063_2022_6401_MOESM7_ESM.pdf]

Charité / 10117 Berlin

Ms.
Univ.-Prof. Dr. med. Claudia Spies
Department of Anesthesiology and
Intensive Care Medicine
Campus Virchow Klinikum
Mittelallee 3, 2.OG, Sekretariat
CVK

Ethics Committee
Ethics Committee at Campus Charité-Mitte

Chairman: Prof. Dr. R. Morgenstern

Management: Dr. med. Katja Orzechowski
ethikkommission@charite.de

Correspondence address: Charitéplatz 1, 10117 Berlin
Tel.: 030/450-517222
Fax: 030/450-517952

http://ethikkommission.charite.de

Cc: kat.schmidt@charite.de

Date: 25th of November, 2019

PRÄP-GO - Prehabilitation of elderly patients with frailty syndrome before elective surgery - Randomized controlled multicenter trial.

Application number: EA1/225/19

Case file from 10/31/2019, received on 10/31/2019, and email dated 10/31/2019.

Dear Prof. Spies,

We hereby acknowledge receipt of your letter dated 10/15/2019 with the following attachments:

- Ethics application form V1.0
- Study Information for Participants and Data Processing V1.0
- Declaration of Consent Form for Participants and Data Processing for healthcare pathway V1.0
- Declaration of Consent Form for Participants and Data Processing Research Purposes V1.0
- Participation information and Consent for Relatives V1.0
- Participant Information NCD control group V1.0
- Declaration of Consent NCD control group V1.0
- Patient Information and Consent Form Descriptive Control V1.0
- Diary for Study Participants V1.0
- Item list V1.0
- Company Liability Insurance of the Charité

The application you submitted was reconsidered by the Ethics Committee CCM of the Ethics Committee at the meeting on 11/14/2019.

The Ethics Committee approves the above-mentioned project subject to the following condition:

In the "Study Information... on participation in the special healthcare pathway, etc.", it should be clearly stated that revocation of participation in the research project is possible at any time, irrespective of revocation of participation in the new healthcare pathway.

Aspects of research projects relating to data protection law are only examined by the ethics committee in a cursory manner. This vote therefore does not replace consultation with the responsible data protection officer.

The ethics committee points out that the ethical and legal responsibility for the implementation of the research project - independent of the result of the ethics committee's consultation - remains with the head of the research project and his staff.

Best regards,

Prof. Dr. med. R. Morgenstern
Chairman

Charité / 10117 Berlin

Ms.
Univ.-Prof. Dr. med. Claudia Spies
Department of Anesthesiology and
Intensive Care Medicine
Campus Virchow Klinikum
Mittelallee 3, 2.OG, Sekretariat
CVK

Ethics Committee
Ethics Committee at Campus Charité-Mitte

Chairman: Prof. Dr. R. Morgenstern

Management: Dr. med. Katja Orzechowski
ethikkommission@charite.de

Correspondence address: Charitéplatz 1, 10117 Berlin
Tel.: 030/450-517222
Fax: 030/450-517952

http://ethikkommission.charite.de

Cc: kat.schmidt@charite.de

Date: 12th of December, 2019

PRÄP-GO - Prehabilitation of elderly patients with frailty syndrome before elective surgery - Randomized controlled multicenter trial.

Application number: EA1/225/19

Case file from 12/02/2019, received on 12/05/2019, and email dated 12/02/2019.

Dear Prof. Spies,

We hereby acknowledge receipt of your letter dated 12/02/2019 with the following attachments:

- Study Information for Participants and Data Processing, V1.1 dated 11/28/2019.
- Declaration of Consent Form for Participants and Data Processing for healthcare pathway, V1.1 of 11/28/2019

The requirements according to the vote of 11/25/2019 are therefore fulfilled. We wish you every success in the implementation of the above study.

Best regards,

Prof. Dr. med. R. Morgenstern
Chairman
