# Supplementary material for: Prehabilitation of elderly frail or pre-frail patients prior to elective surgery (PRAEP-GO): study protocol for a randomized, controlled, outcome assessor-blinded trial
Source: Trials. 2022 Jun 6;23:468. doi: 10.1186/s13063-022-06401-x (PMC9167908; doi:10.1186/s13063-022-06401-x)
Supplement: Supplementary file 6 — Additional file 6. Grant decision letter. [file 13063_2022_6401_MOESM6_ESM.pdf]

From: Gemeinsamer Bundesausschuss – Innovationsausschuss
Wegelystr. 8 10623 Berlin
Contact at DLR-PT: Dr. Bettina Möller-Bock
Phone: +49 228 3821-1240
Telefax: +49 228 3821-1257
E-Mail: bettina.moeller-bock@dlr.de

To: Charité-Universitätsmedizin Berlin
Geschäftsbereich Forschung
Drittmittelantragsservice (DAS)
Frau Marion Herrmann
Charitéplatz 1
10117 Berlin

9th of January, 2019

Notice of Funding

Funding from the Innovation Fund for the promotion of new healthcare pathways (§ 92a para. 1 SGB V) for the project:
"PRECO-Frail - Preconditioning of the elderly surgical patient with frailty".
Grant number: 01NVF18024

Executing agency: Department of Anesthesiology and Intensive Care, Campus Virchow-Klinikum and Campus Mitte

Project management: Prof. Dr. Claudia Spies

Your application was received on March 20, 2018
with supplements dated March 21, 2018, November 5, 2018, and November 6, 2018 (e-mail).

Dear Ms. Herrmann,

Based on the decision of the Innovation Committee of October 18, 2018, this funding decision grants the following project funding from the Innovation Fund. This funding decision is at the same time an act of entrustment within the meaning of the SGEI exemption decision listed below.

I. Entrustment

Pursuant to European Commission Decision 2012/21/EU of 20 December 2011 on the application of Article 106(2) of the Treaty on the Functioning of the European Union (TFEU) to State aid in the form of public service compensation granted to certain enterprises entrusted with the operation of services of general economic interest (OJ. EU L 7/3 of January 11, 2012, "SGEI Exemption Decision"), the compatibility with the internal market of compensation for costs incurred by an enterprise for the provision of services of general economic interest ("SGEI") requires, among others, a proper act of entrustment within the meaning of Article 4 of the SGEI Exemption Decision.

It is established that the implementation of the project of Charité-Universitätsmedizin Berlin entitled "PRECO-Frail - Preconditioning of the elderly surgical patient with frailty" is a service of general economic interest. The project contributes to the further development of care in the statutory health insurance pursuant to Section 92a (1) SGB V. With the present funding decision, Charité-Universitätsmedizin Berlin is entrusted to provide this service in accordance with its application received on March 20, 2018, with the above-mentioned additions, during the funding period from July 1, 2019, to June 30, 2022.

The monitoring and modification of the compensation services in the form of the granted subsidies results from ANBest-IF (Annex N). The result of the calculation of the compensation services will be set forth in a notice of amendment after review of the revised financing plan requested in accordance with item III. 2. The use of the approved funding may not exceed what is necessary to cover the expenses caused by the fulfillment of the public service obligation. In this context, reference is made to No. 3 ANBest-IF.

The monitoring and avoidance of a possible overcompensation payment is regulated in No. 7 and No. 14 ANBest-IF. The reclaim results from No. 19 ANBest-IF.

These provisions also apply to consortium partners in the event that parts of the funding are passed on.

II. Purpose of funding, scope of funding and payment schedule

On the basis of your application received on March 20, 2018, with the above-mentioned additions, you are granted funding pursuant to Section 92a (1) of the German Social Code, Book V (SGB V) and the Rules of Procedure of the Innovation Committee at the Federal Joint Committee for the period

from July 1, 2019 to June 30, 2022 (funding period)

a non-repayable, **full funding** amount of

up to **€8,724,270.00**
(in letters: eight-seven-two-four-two-seven-zero-comma-zero-zero euros),

but no more than the amount of the eligible expenses.

The subsidies are earmarked for a specific purpose and may only be settled for the expenses incurred for the above-mentioned project during the subsidy period.

Approval is subject to the condition that the overall financing of the project remains secured.

III. Additional provisions

The attached General Auxiliary Provisions of the Innovation Committee of the Joint Federal Committee for Grants from the Innovation Fund (ANBest-IF, Annex N) and the following special auxiliary provisions form an integral part of this funding decision:

1. Entrusted enterprise∗ [∗ Applies to all consortium partners in accordance with No. 4 Sentence4.]

As an entrusted enterprise as defined in Item I of this funding decision, the Recipient must report the expenditures and revenues related to the SGEI separately from all other activities. To this end, compensation in the form of awarded grant funds must be managed in a separate project account. In

addition, all expenses actually incurred for the provision of the SGEI must be substantiated by means of personnel records and receipts.

2. Condition for the effectuality of the funding decision*.

The funding is approved subject to a condition precedent (Section 32 (2) No. 2 of the Tenth Book of the German Social Code, SGB X). The condition precedent is fulfilled if the following documents are submitted in coordination with the DLR Project Management Agency and their review leads to a positive result.

- Submission of a revised financing plan for both the consortium management and the consortium partners, including explanations and calculation bases,
- Submission of an updated costing sheet (Annex 4 to the project description)
  Please note the following:
  The use of the requested funds must be described in detail in the calculation sheet. The information must be comprehensible and understandable for third parties without further research. Additional sheets may need to be attached to explain or justify the individual expenses. The amounts for the use of funds must be comprehensibly derived from the financing plan. In order to clarify the calculation method, formulas and, if necessary, an additional sheet should be used as a secondary calculation. If the expenditures for health care services include investments, these must be broken down transparently. It must also be explained for each service why it is not covered by standard care. Performance-related remuneration and billing must be ensured. Expenditures for health care services are only eligible for funding if the health care services provided are also included in the scientific monitoring and evaluation.
- The respective existing financing responsibilities of providers and institutions outside the statutory health insurance system remain unaffected. This must be taken into account when revising the financing plan.
- Submission of a more detailed evaluation concept including a graphical representation of the course of the study (e.g. CONSORT flow chart) with the signature of the responsible methodologist. In particular, the methodology, the intervention and control group as well as the planning of case numbers are to be presented in more detail.
- Submit an updated and more detailed timeline for case number achievement according to the attached form (Attachment P),
- The project structure is to be explained in more detail. The allocation of certain described tasks within the consortium partnership is not comprehensible. In particular, with regard to the two consortium partners Irmgard Landgraf - Practice for Internal Medicine and Prof. Schwantes Practice, it must be explained why their tasks cannot be carried out by the consortium management or as part of a subcontract.
- It must also be explained how access to the outpatient sector is provided. In this context, a statement must also be submitted showing how case-payment attainment is to be ensured.
- A project title in German must be chosen.

The legally binding signed documents must be sent to the DLR Project Management Agency **by February 28, 2019 at the latest**. This funding decision will only become effective once the positive review of the aforementioned documents has been confirmed by the funding agency. A corresponding notification of amendment will be issued for this purpose.

3. Milestone plan

The Recipient shall submit a Milestone Plan for the Project by **February 28, 2019**, in accordance with the attached form (Attachment M).

The Milestone Plan must generally include at least one relevant milestone in each quarter of the project period. A current Gantt chart for work and schedule planning must also be submitted with the Milestone Plan. The milestone plan, Gantt chart, and caseload achievement schedule must match without contradiction. The representation of the quarters in the Gantt chart must refer to the years of the project duration, so that the project progress and the milestone achievement are comprehensible.

4. Forwarding of grant funds

The Recipient is authorized to forward parts of the funding to the consortium partners named in the above application with the above supplements.

The provisions of No. 1 ANBest-IF and Annex W must be observed. The additional provisions marked with * in this funding decision are binding for all consortium partners concerned.

5. E-health solutions/Telemedicine*.

According to Section 291d SGB V, the relevant specifications must be observed when using information technology systems and, in particular, open interfaces and interoperability must be ensured. When using electronic applications, the regulations on the interoperability directory in accordance with Section 291e (10) SGB V must be taken into account.

Please note that the Gesellschaft für Telematik (Society for Telematics) has bindingly defined the secure procedures for transmitting medical documents via the telematics infrastructure in accordance with Section 291b (1e) SGB V (https://fachportal.gematik.de/spezifikationen/sichere-uebermittlungsverfahren/). The funding recipient must check whether the project is affected and, if necessary, make adjustments.

For the use of other electronic health care applications and health research applications that go beyond the applications of the electronic health card (eGK), the requirements of § 291a Para. 7 Sentence 3 SGB V and the gematik usage requirements of § 291b Para. 1b Sentence 3 SGB V must also be met (see gematik-homepage https://fachportal.gematik.de/fileadmin/user_upload/fachportal/files/Spezifikationen/Weitere-Anwendungen/gemRL_NvTIwA_V1.3.0.pdf). The confirmation procedure with gematik (Society for Telematics Applications of the Health Card) in accordance with § 291b Para. 1b Sentence 4 SGB V must be completed by the start of the project at the latest.

6. Data protection*

The legal framework for processing personal patient data, which results in particular from the provisions of the General Data Protection Regulation, the Federal Data Protection Act (BDSG), SGB V and SGB X, must be observed. In cases of doubt, the authorities responsible for data protection are to be consulted.

7 Ethical Guidelines*

The recommendations of the Declaration of Helsinki as well as the guidelines of the CIOMS (Council for International Organization of Medical Sciences) and the WHO (World Health Organization): "Proposed International Guidelines For Biomedical Research Involving Human Subjects" in the respective valid versions are to be adhered to when conducting investigations on humans and/or obtaining or using human sample material within the scope of this project.

8. Ethics vote*

Prior to the start of research involving human subjects and/or the collection or use of human sample material, the unqualified positive vote of the responsible ethics committee must be submitted. If the committee does not consider a positive vote to be necessary, a statement to this effect must be submitted to the ethics committee.

9. Reservation of revocation*.

This notice may be revoked and funding may be discontinued in whole or in part in one of the following cases (reservation of revocation pursuant to Section 32 (2) No. 3 in conjunction with Section 47 (1) No. 1 SGB X):

- in the event that the funding purpose cannot be achieved,
- in the case of a blocking of funds for certain approaches of the financing plan,
- in the cases of non-timely or incomplete submission of evidence,
- for compelling reasons.

10. Proof of use*

The regulations of No. 14 ANBest-IF apply to the proof of use. The relevant forms will be made available to the Recipient in due course. For the list of receipts, the form according to Annex B is to be used.

11. Cooperation with external partners without own funding*.

For the following external cooperation partner, which is essential for the implementation of the project, a meaningful letter of intent signed by the person responsible for the cooperation must be submitted by **February 28, 2019**:

- Vivantes Klinikum Spandau

12. Project events

The sponsor is to be informed about or invited to important dates or events, newsletters, etc. Upon request, information on the type, scope and success of the measures carried out must be provided at any time.

13. Distribution of funds

Funding will be disbursed upon request by the Recipient in accordance with No. 7 ANBest-IF. Project-related eligible expenses incurred after the start of funding can be submitted later with the payment request for settlement.

Funding cannot be paid out until the notice of award has become final after expiration of the time limit for appeal, the condition for the effectiveness of the notice of award has been fulfilled in accordance with Item III. 2 of this notice of award, and the confirmation of receipt (form in accordance with Attachment E) has been submitted. The Recipient may bring about the validity of the decision beforehand by waiving the right to appeal (form in Annex E).

For the preparation of the payment request, the form according to Annex Z is to be used and Annex H is to be taken into account. For the status report to be submitted with the payment request (see No. 7 ANBest-IF), the form according to Annex S is to be used.

In addition, the current status of caseload achievement for the project must be reported with each payment request using the attached form (Attachment P).

Consortium partners shall use the form provided in Attachment K to request funds from consortium leadership.

14. Repayments

Repayments of grant funds, as well as interest, if any, shall be made to the bank account below, quoting grant number 01NVF18024:

> Account holder: Federal Joint Committee
> Bank: Deutsche Apotheker- und Ärztebank eG
> IBAN: DE69 3006 0601 0004 2118 20
> BIC: DAAEDEDDXXX

15. Commissioning of a project sponsor

As the project management organization for the Innovation Committee at the Federal Joint Committee, the DLR Project Management Organization is currently commissioned to handle project funding within the framework of the Innovation Fund.

All transactions concerning the implementation and processing of the project are to be sent to the project management organization:

DLR Project Management Agency
- Health Division -
Heinrich-Konen-Str. 1
53227 Bonn, Germany

<u>Technical support:</u>
Dr. Bettina Möller-Bock
Tel.: +49 228 3821-1240
E-mail: bettina.moeller-bock@dlr.de

<u>Administrative support:</u>
Simone Cabalo
Tel.: +49 228 3821-1668
E-mail: simone.cabalo@dlr.de

A **copy** of the notification will be sent to the project management and the administrative contact by e-mail.

Yours sincerely,

Prof. Josef Hecken
Dieser Bescheid wurde elektronisch erstellt und ist auch ohne Originalunterschrift gültig.

[Attachments]

Information on legal remedies

An appeal against this funding decision may be filed within one month of notification with the Berlin-Brandenburg Regional Social Court, Försterweg 2-6, 14482 Potsdam, Germany, in writing or on the record of the clerk of the court. The action may also be filed in accordance with the provisions of the Ordinance on Electronic Legal Transactions in the State of Brandenburg of June 14, 2006 (GVBl.II/06, [No. 33], p. 558), as amended on December 19, 2017 (GVBl.II/17, [No. 73]), using a qualified electronic signature in electronic form at the electronic mailroom of the Berlin-Brandenburg Regional Social Court via the communication channels designated on the website www.erv.brandenburg.de.
